# Cell division factor ZapE regulates *Pseudomonas aeruginosa* biofilm formation by impacting the *pqs* quorum sensing system

**DOI:** 10.1002/mlf2.12059

**Published:** 2023-03-21

**Authors:** Xi Liu, Minlu Jia, Jing Wang, Hang Cheng, Zhao Cai, Zhaoxiao Yu, Yang Liu, Luyan Z. Ma, Lianhui Zhang, Yingdan Zhang, Liang Yang

**Affiliations:** ^1^ State Key Laboratory of Microbial Resources, Institute of Microbiology Chinese Academy of Sciences Beijing China; ^2^ Key University Laboratory of Metabolism and Health of Guangdong, School of Medicine Southern University of Science and Technology Shenzhen China; ^3^ Guangdong Province Key Laboratory of Microbial Signals and Disease Control, Integrative Microbiology Research Center South China Agricultural University Guangzhou China; ^4^ Medical Research Center Southern University of Science and Technology Hospital Shenzhen China; ^5^ Shenzhen Third People's Hospital, National Clinical Research Center for Infectious Disease The Second Affiliated Hospital of Southern University of Science and Technology Shenzhen China

**Keywords:** biofilm, *Pseudomonas aeruginosa*, *Pseudomonas* quinolone signal, transposon‐insertion site sequencing (Tn‐seq), ZapE

## Abstract

*Pseudomonas aeruginosa* is one of the leading nosocomial pathogens that causes both severe acute and chronic infections. The strong capacity of *P. aeruginosa* to form biofilms can dramatically increase its antibiotic resistance and lead to treatment failure. The biofilm resident bacterial cells display distinct gene expression profiles and phenotypes compared to their free‐living counterparts. Elucidating the genetic determinants of biofilm formation is crucial for the development of antibiofilm drugs. In this study, a high‐throughput transposon‐insertion site sequencing (Tn‐seq) approach was employed to identify novel *P. aeruginosa* biofilm genetic determinants. When analyzing the novel biofilm regulatory genes, we found that the cell division factor ZapE (PA4438) controls the *P. aeruginosa pqs* quorum sensing system. The ∆*zapE* mutant lost fitness against the wild‐type PAO1 strain in biofilms and its production of 2‐heptyl‐3‐hydroxy‐4(1H)‐quinolone (PQS) had been reduced. Further biochemical analysis showed that ZapE interacts with PqsH, which encodes the synthase that converts 2‐heptyl‐4‐quinolone (HHQ) to PQS. In addition, site‐directed mutagenesis of the ATPase active site of ZapE (K72A) abolished the positive regulation of ZapE on PQS signaling. As ZapE is highly conserved among the *Pseudomonas* group, our study suggests that it is a potential drug target for the control of *Pseudomonas* infections.

## Impact statement

The drug‐resistant biofilm‐associated infections caused by *Pseudomonas aeruginosa* pose critical challenges for clinical treatment, calling for novel antimicrobial strategies targeting biofilm formation mechanisms. Previous investigations of biofilm formation revealed the critical regulatory roles of quorum sensing. In this study, we applied a high‐throughput method to identify novel genetical determinants of *P. aeruginosa* biofilm formation. We found ZapE (PA4438), a cell division factor and an ATPase, that played an important role in regulating *P. aeruginosa* biofilm formation through the *pqs* quorum sensing system. Our findings provide a collection of data supporting the concept that ZapE could be a feasible target for antibiofilm and antivirulence approaches.

## INTRODUCTION

*Pseudomonas aeruginosa* is a notorious opportunistic human pathogen causing a wide range of infections, such as cystic fibrosis (CF) lung infections, ventilator‐associated pneumonia, and chronic wound infections^1^, ^2^. *P. aeruginosa* infections are difficult to treat due to their increasing resistance as well as their biofilm formation capacity. Biofilms impede the diffusion of antimicrobial agents and preserve virulence factors (e.g., rhamnolipids), which impairs host defense systems and leads to persistent infections^3^, ^4^. Moreover, the high cell density within the biofilm also promotes an ideal environment for the adaptive evolution of novel bacterial lineages such as the small colony variants^5^.

Biofilm cells have a distinct physiology compared to their free‐living counterparts^6^. Multiple signaling pathways participate in the regulation of biofilm development, such as synthesis of extracellular polymeric substances (EPS), downregulation of motility, and enhancement of adhesion. There have been extensive studies focusing on the synthesis, localization, and functions of *P. aeruginosa* exopolysaccharides during biofilm development^7^. The exopolysaccharide synthesis in *P. aeruginosa* as well as many other bacteria is under the control of bis‐(3'‐5')‐cyclic diguanylic acid (c‐di‐GMP), a widely distributed bacterial intracellular secondary messenger that also regulates many other physiological processes^8^. C‐di‐GMP regulates its target pathways via specific effectors such as PilZ domain containing proteins, which can then directly or indirectly affect gene expression^8^. The bacterial cell‐to‐cell communication system (also known as quorum sensing, QS) is another essential regulatory mechanism for biofilm formation^9^. QS employs small diffusible signal molecules to coordinate the expression of genes in a density‐dependent manner and is, therefore, an ideal strategy to regulate gene expression in the biofilm microcolonies.

*P. aeruginosa* has four QS systems, *las*/*rhl*/*pqs*/*iqs*, that regulate a large set of genes in its genome^10^. The *las* and *rhl* QS systems employ *N*‐acyl‐homoserine lactones (AHLs) as signaling molecules, which activate specific intracellular LuxR‐type receptors to regulate gene expression. The third QS system of *P. aeruginosa* uses the *Pseudomonas* quinolone signal (*pqs*), heptyl‐3‐hydroxy‐4‐(1H)‐quinolone (PQS) as a QS molecule, which is highly specific to *Pseudomonas* species and could serve as a biomarker for the diagnosis of *P. aeruginosa* infections^10^, ^11^, ^12^. The *pqs* QS system is more complicated than the AHL QS systems in that the chemical compound 2‐heptyl‐4‐quinolone (HHQ) is synthesized by the *pqsABCD* gene cluster and converted to PQS via a monooxygenase PqsH^10^, ^11^, ^13^. *pqs* QS regulates the biosynthesis of virulence factors such as pyocyanin and the PQS could serve as an iron chelator. Iron deficiency condition stimulates PQS synthesis, whereas excessive production of PQS will lead to autolysis of *P. aeruginosa* and the release of extracellular DNA (eDNA), which is an important biofilm matrix component^14^, ^15^. Currently, the induction kinetics and function of the *pqs* QS system are still not fully elucidated.

Transposon insertion site sequencing (Tn‐seq), which combines the transposon mutagenesis method with high‐throughput sequencing technology, is a global approach to provide comprehensive information on gene function on a genome‐wide scale^16^, ^17^. Himar1, a transposon that could insert randomly at TA dinucleotides^18^, has been previously used in *P. aeruginosa* investigations. By applying Tn‐seq analysis on tubing‐biofilms, five novel genetic determinants were identified for *P. aeruginosa* biofilm formation in this study. Among them, we systematically investigated the role of PA4438. PA4438 is predicted to be homologous to *zapE*, which encodes a cell division factor and AFG1 (ATPase family gene 1, which belongs to AAA+ protein) in *Escherichia coli* ^19^. Moreover, we observed that the ∆PA4438 mutant had a similar elongated phenotype under oxygen limitation and high‐temperature conditions as the *E. coli* ∆*zapE* mutant, hence, we named PA4438 as *zapE*. We provide evidence that ZapE is involved in the conversion of HHQ to PQS by PqsH, which is dependent on its ATP hydrolyzing activity. Based on our study, we speculate that the AAA+ protein ZapE is a potential drug target due to its multiple functions involved in both cell division and PQS signaling.

## RESULTS

### Tn‐seq analysis of *P. aeruginosa* biofilm formation

To systematically investigate genes that contribute to *P. aeruginosa* biofilm formation, Tn‐seq analysis was employed to study *P. aeruginosa* tubular biofilms. A pooled library of nearly 500,000 mutants was constructed in the wild‐type strain PAO1 by using the Himar1 transposon. In this PAO1 mutant library, the coverage rate of TA insertion sites was about 55%, which was higher than the ideal coverage of 30%, suggesting that coverage of TA insertion sites reached sufficient saturation. After the bottom 10% read counts were removed, there were 5158 insertion genes in this library (compared to the 5570 genes in the PAO1 genome), which further confirmed that the library contained almost all nonessential gene mutants.

**Figure 1:**
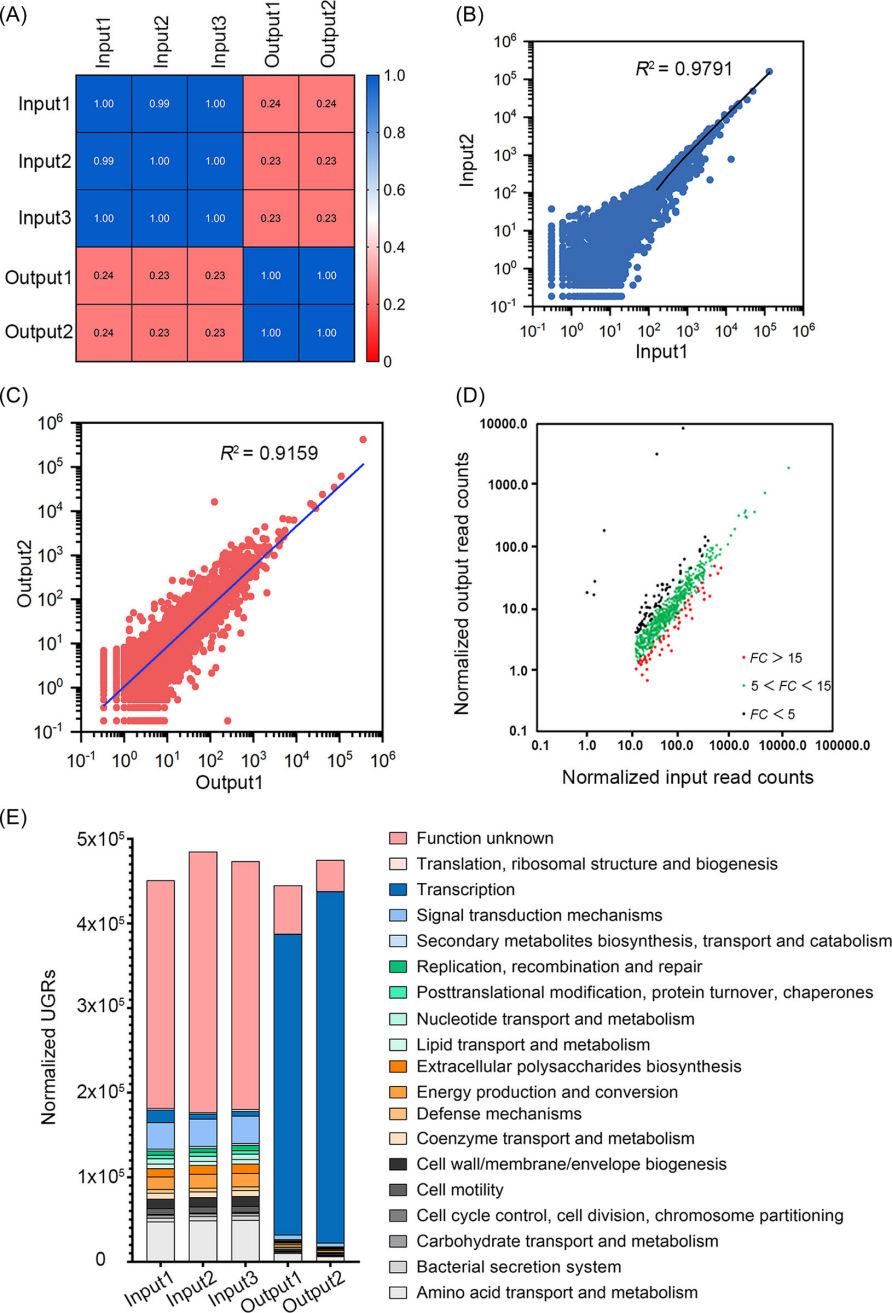
Transposon‐insertion site sequencing analysis of genetic determinants for *Pseudomonas aeruginosa* biofilm formation. (A) Correlation analysis of input and output samples. (B) Quality control analysis of two inputs from the same batch of experiments. Each point represents the number of unique gene reads (UGRs) of one specific mutagenized gene in the duplicate samples. (C) Quality control analysis of two outputs from different batches of experiments. (D) Dot plot showing the significantly reduced colonization of mutants during biofilm formation. Each dot represents the normalized UGRs of each gene in input and output. Fold change (*FC*) is colorized to show the differences. (E) Functional categories of essential genes during biofilm formation and maintenance. Genes were deemed as essential factors during *P. aeruginosa* biofilm formation, in the scenario that all mutants impart no fitness cost, when log_2_|*FC* (input/output)| > 2 and *p* < 0.05.

As shown in the schematic drawing in Figure S1A, the PAO1 mutant library was cultivated in the tubular biofilm reactors for 3 days, giving sufficient time to enrich the mutants with good fitness in the biofilm mode of growth, while low fitness biofilm mutants would be selected out. Pearson's correlation analysis indicated that there were significant differences between the input pools and output pools (Figure 1A). Furthermore, the excellent correspondence between technical replicates (*R* ^2^ of two input mutant pools was 0.9791) (Figure 1B) and between biological replicates (*R* ^2^ of two output mutant pools was 0.9159) (Figure 1C) suggested that the cultivation of Tn‐insertion mutants in tubular biofilms with the fluid flow was reproducible.

### Identification of genes required for *P. aeruginosa* biofilm formation

We normalized the abundance of Tn insertion mutants in each mutant pool by DESeq2 and used the normalized values to calculate the input‐to‐output ratio. A high ratio suggests that the corresponding mutated gene is important for biofilm formation. In total, 924 mutants had a statistically different abundance after biofilm cultivation in the tubular system compared to that before cultivation, with 902 mutants having an increase in the input‐to‐output ratio after biofilm cultivation (Figure S1B, Table S1). About 71.0% (640 mutants) of the above mutants had a fivefold or greater reduction in output mutant pools compared to the input mutant pool (Figure 1D), suggesting that the mutated genes might be biofilm determinants. Next, we categorized mutants with high input‐to‐output ratios by gene function and found that the mutated genes were involved in amino acid metabolism, energy metabolism, cell membrane formation, and well‐known biofilm formation mechanisms (Figure 1E). In addition, mutants with insertions in exopolysaccharide synthesis and motility genes also had high input‐to‐output ratios (Figure S2A–E), suggesting that our Tn‐seq results reasonably reflected the previous biofilm studies. Interestingly, besides the above mutants, we also found that mutants with insertions in hypothetical genes had high input‐to‐output ratios, indicating that genes with unknown functions might play important roles in *P. aeruginosa* biofilm formation.

### Validation of candidate biofilm determinants with unknown function

**Figure 2:**
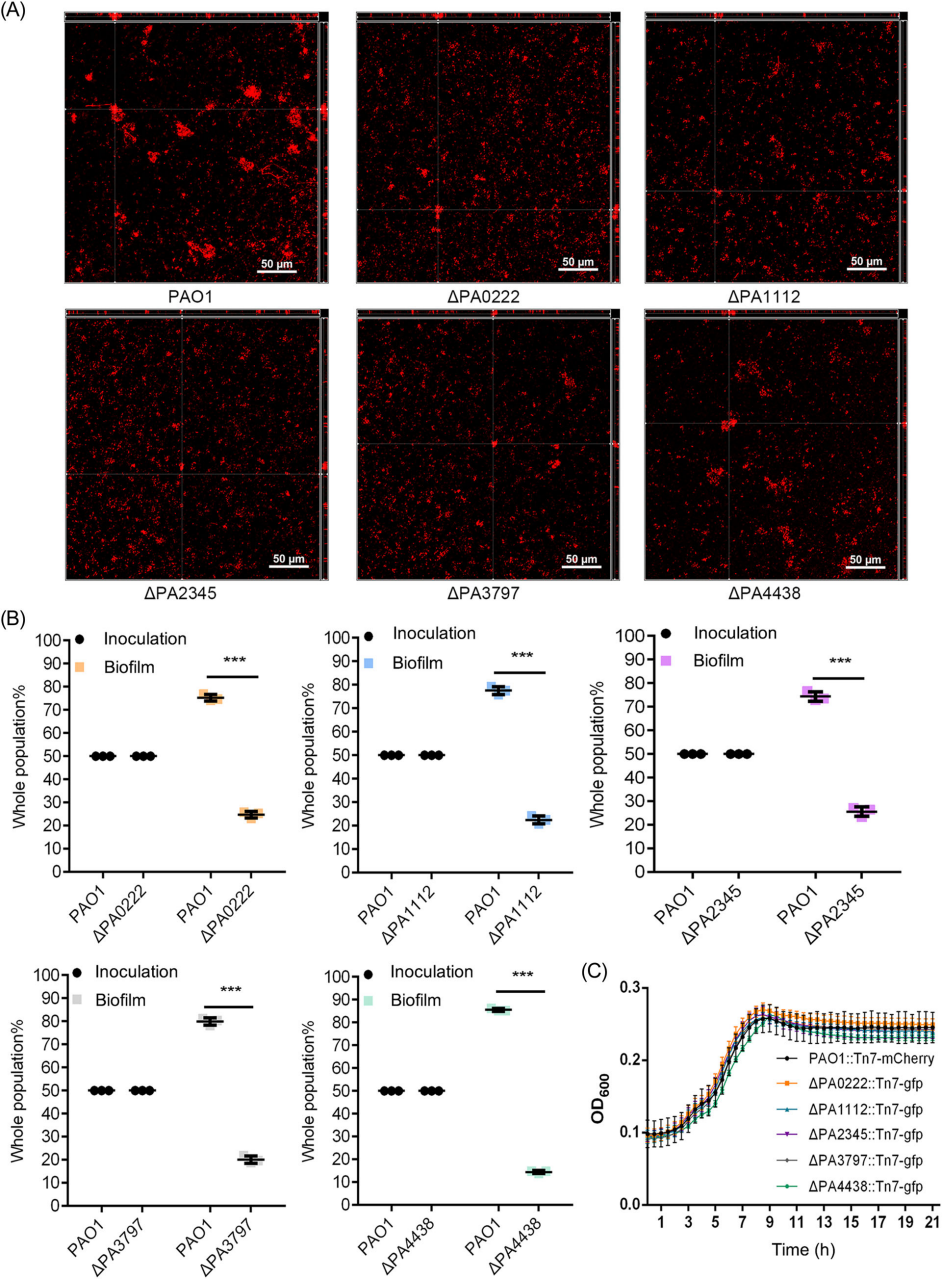
Biofilm formation by the mutants of five candidate biofilm determinants (PA0222, PA1112, PA2345, PA3797, and PA4438) with unknown functions compared to wild‐type strain PAO1. (A) Confocal laser scanning microscope images showing biofilms formed by PAO1, ΔPA0222, ΔPA1112, ΔPA2345, ΔPA3797, and ΔPA4438. Biofilms were stained by SYTORED17. Scale bar = 50 μm. (B) Proportion of PAO1 (tagged with mCherry) and mutants (tagged with GFP) in cocultured biofilms. ***p < 0.001. (C) Growth curve of PAO1, ΔPA0222, ΔPA1112, ΔPA2345, ΔPA3797, and ΔPA4438.

We selected five hypothetical genes (PA0222, PA1112, PA2345, PA3797, and PA4438) with the highest fold changes between input and output mutant pools for experimental validation (Table 1). Clean knockout mutants for these five genes were constructed, and the biofilm morphology of each mutant was examined by using a confocal laser scanning microscope (CLSM). Compared to the PAO1 wild‐type strain, ΔPA0222 showed similar biofilm structures with slightly smaller microcolonies; ΔPA1112, ΔPA2345, and ΔPA3797 formed tiny microcolonies with significantly less biofilm biomass; and ΔPA4438 formed loose biofilm aggregates with decreased amounts of biomass (Figures 2A and S2F). To further compare the biofilm formation ability of different strains, mutants (tagged with GFP) and PAO1 (tagged with mCherry) were mixed at a 1:1 ratio to cultivate biofilms, and flow cytometry was then used to analyze the proportion of fluorescent cells isolated from coculture biofilms. The results show that all five mutants lost fitness against PAO1 in coculture biofilms, and ΔPA4438 had the lowest fitness among the five mutants (Figure 2B). Furthermore, the growth rates of these five mutants showed no significant difference compared to PAO1 (Figure 2C), suggesting that the reduced biofilm biomass and fitness of these mutants in mixed biofilms were not due to growth deficiency. Taken together, these results suggest that each of the five genes, particularly PA4438, plays an important role in *P. aeruginosa* biofilm formation and are novel genetic determinants.

**Table 1:** Hypothetical genes chosen in this study.

Gene ID	*FC* (In/Out)	Predicted product/function	UniProt ID
PA0222	16.95	Quorum sensing, bacterial extracellular solute‐binding protein.	Q9I6R6_PSEAE
PA1112	16.28	GSDH domain‐containing protein; glucose/sorbosone dehydrogenase	Q9I4M3_PSEAE
PA2345	9.49	Oxidation–reduction process, sulfur metabolism; flavin adenine dinucleotide (FAD) binding.	Q9I1D3_PSEAE
PA3797	10.16	Nitrogen compound metabolic process, carbon–nitrogen hydrolase superfamily.	Q9HXK0_PSEAE
PA4438	14.26	Cell division protein ZapE; cell division, ATPase activity, AFG1‐like ATPase.	Q9HVX7_PSEAE

*FC*, fold change; In, input; Out, output.

### PA4438 is a homologue of the *E. coli* cell division protein ZapE

**Figure 3:**
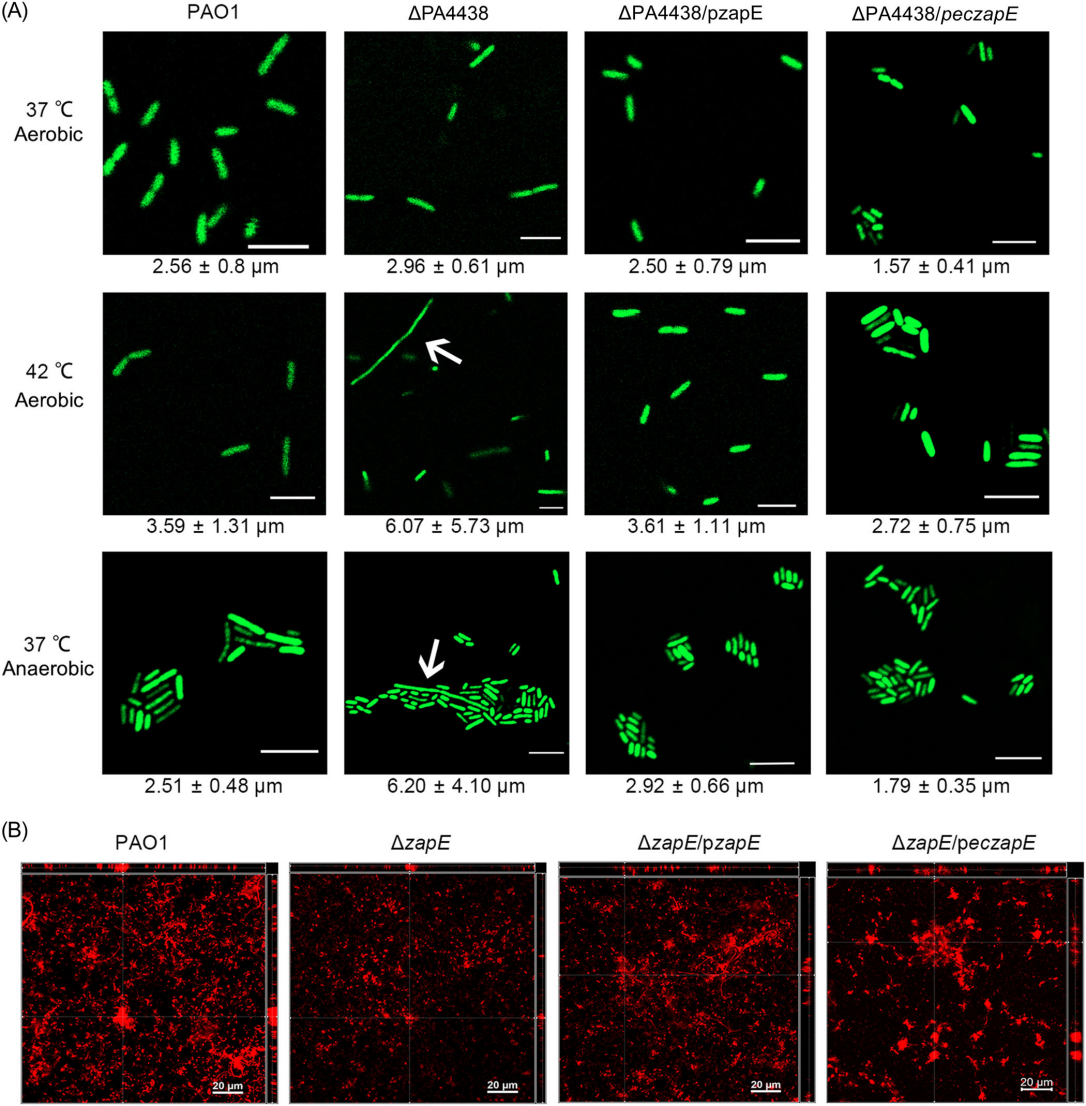
ZapE is required for proper cell division under high‐temperature and oxygen‐limited conditions and biofilm formation. (A) Morphology of PAO1, ΔPA4438, its *zapE* complementation strain ΔPA4438/p*zapE* and *Escherichia coli zapE* complementation strain ΔPA4438/p*eczapE* were cultivated in 37°C aerobic, 42°C aerobic, and 37°C anaerobic environment. The white arrows point to the elongated cells. Scale bar = 5 μm. (B) Biofilm morphology of PAO1, Δ*zapE*, Δ*zapE*/p*zapE*, and Δ*zapE*/p*eczapE* under 37°C aerobic condition. Biofilms were stained by SYTORED17. Scale bar = 20 μm.

PA4438 is predicted to encode an AAA+ AFG1 ATPase and its predicted amino acid sequence has regions of high conservation to the cell division protein ZapE of *E. coli* (EcZapE) (Figure S3). A previous study reported that the loss of EcZapE would result in cell elongation under oxygen‐limited and high‐temperature conditions^19^. We examined the morphology of PAO1, ΔPA4438, and its complementation strains under 37°C aerobic, 42°C aerobic, and 37°C anaerobic environment conditions. Significant elongation of ΔPA4438 cell size was observed under 42°C aerobic and 37°C anaerobic conditions (Figure 3A). Complementation by PA4438 or *eczapE* (*zapE* from *E. coli*) to the ΔPA4438 mutant (ΔPA4438/pPA4438 and ΔPA4438/p*eczapE*) reverted its cell size back to a similar length of PAO1 under anaerobiosis and high‐temperature conditions (Figure 3A). These results confirmed that PA4438 encodes a functional homologue of EcZapE, hereafter, we name PA4438 as *zapE*.

Next, we evaluated the biofilm formation ability of PAO1, Δ*zapE*, Δ*zapE*/p*zapE*, and ∆*zapE*/p*eczapE* (Figure 3B). The biofilm phenotype of the two complementation strains showed no significant difference with PAO1, implying that EcZapE might play a regulatory role in *E. coli* biofilm formation. This suggests that *zapE* (PA4438) is not only important for regulating biofilm formation but also for proper cell division under oxygen‐limited and high‐temperature environments.

### *ZapE* regulates the *pqs* QS and pyoverdine synthesis

**Figure 4:**
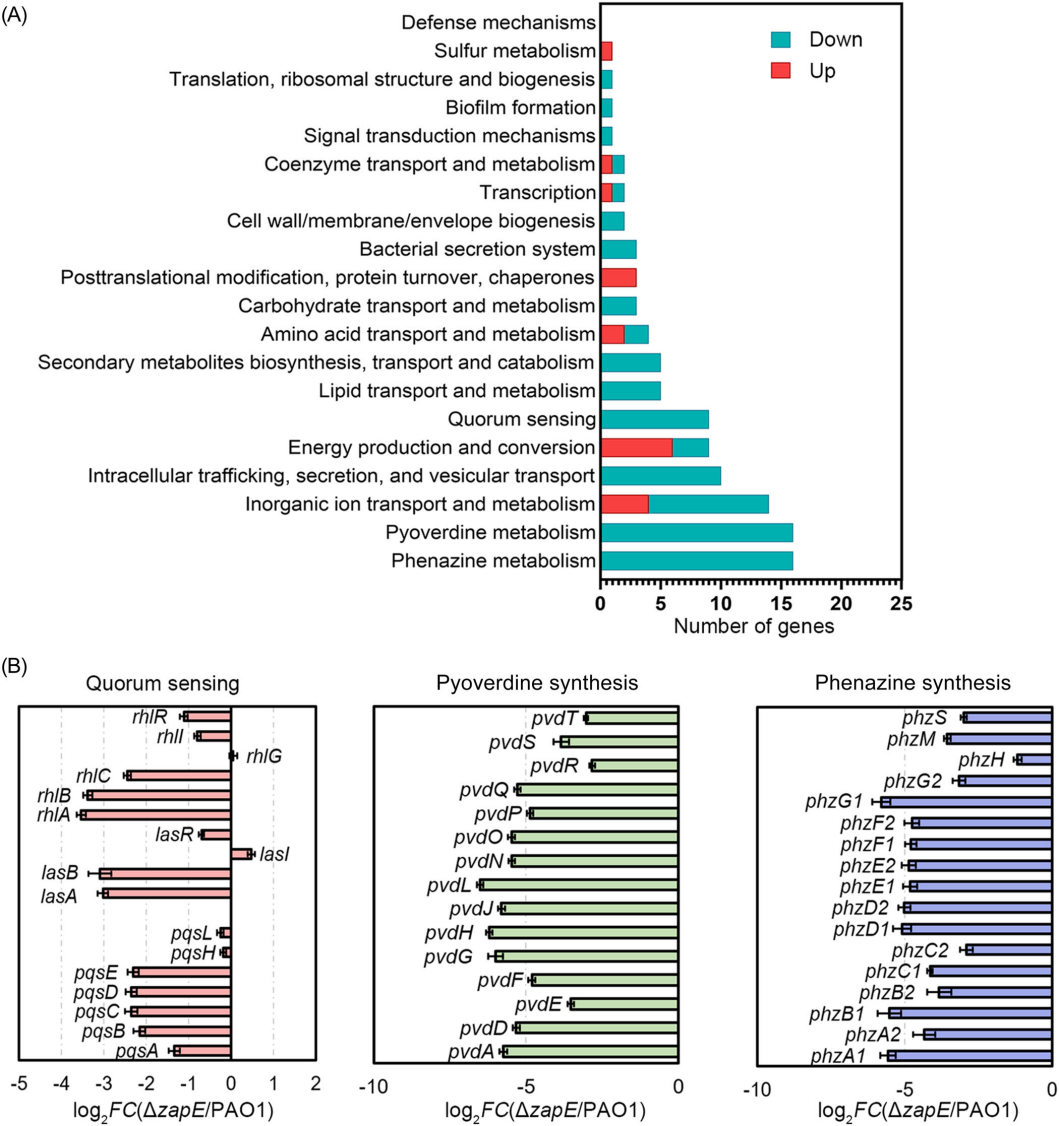
Expression of genes related to phenazine synthesis, pyoverdine synthesis, and quorum sensing is significantly downregulated in Δ*zapE* compared to PAO1. (A) Functional categories of significantly dysregulated genes (log_2_|*FC* (Δ*zapE*/PAO1)| > 2 and *p*< 0.05). (B) Dysregulated genes involved in quorum sensing, pyoverdine synthesis, and phenazine synthesis.

To further investigate the regulatory mechanism of *zapE* in *P. aeruginosa* biofilm formation, the EPS production and motility of the Δ*zapE* mutant were examined. The results showed that exopolysaccharides and motility of Δ*zapE* were indistinguishable from PAO1 (Figure S4). Next, RNA‐sequencing‐based transcriptomic analysis was employed to elucidate the global effect of *zapE* on *P. aeruginosa* gene expression. This analysis showed that 277 genes were differently expressed in the Δ*zapE* compared to PAO1 (log_2_|Fold‐change| > 2, *p* <  0.05), including 37 upregulated genes and 240 downregulated genes (Figure S5A). Among these 277 significant genes, there were 111 genes with annotated functions. The expression levels of genes that encode well‐known biofilm structural factors such as exopolysaccharides, pili, and flagellar were similar between Δ*zapE* and PAO1 (Figure S5B–E), which is consistent with the results from phenotypic analysis (Figure S4). Strikingly, the expression levels of genes involved in phenazine synthesis (14.4% of the total differentially expressed genes with annotated function), pyoverdine synthesis (14.4% of the total differentially expressed genes with annotated function), inorganic ion transport and metabolism (12.6% of the total differentially expressed genes with annotated function), intracellular trafficking and secretion (9.0% of the total differentially expressed genes with annotated function), energy metabolism (8.1% of the total differentially expressed genes with annotated function), and QS (8.1% of the total differentially expressed genes with annotated function) showed the biggest difference between Δ*zapE* and PAO1 (Figure 4A). Almost all *pqs* QS regulated genes were expressed at a lower level in Δ*zapE* compared to PAO1 (Figure 4B).

**Figure 5:**
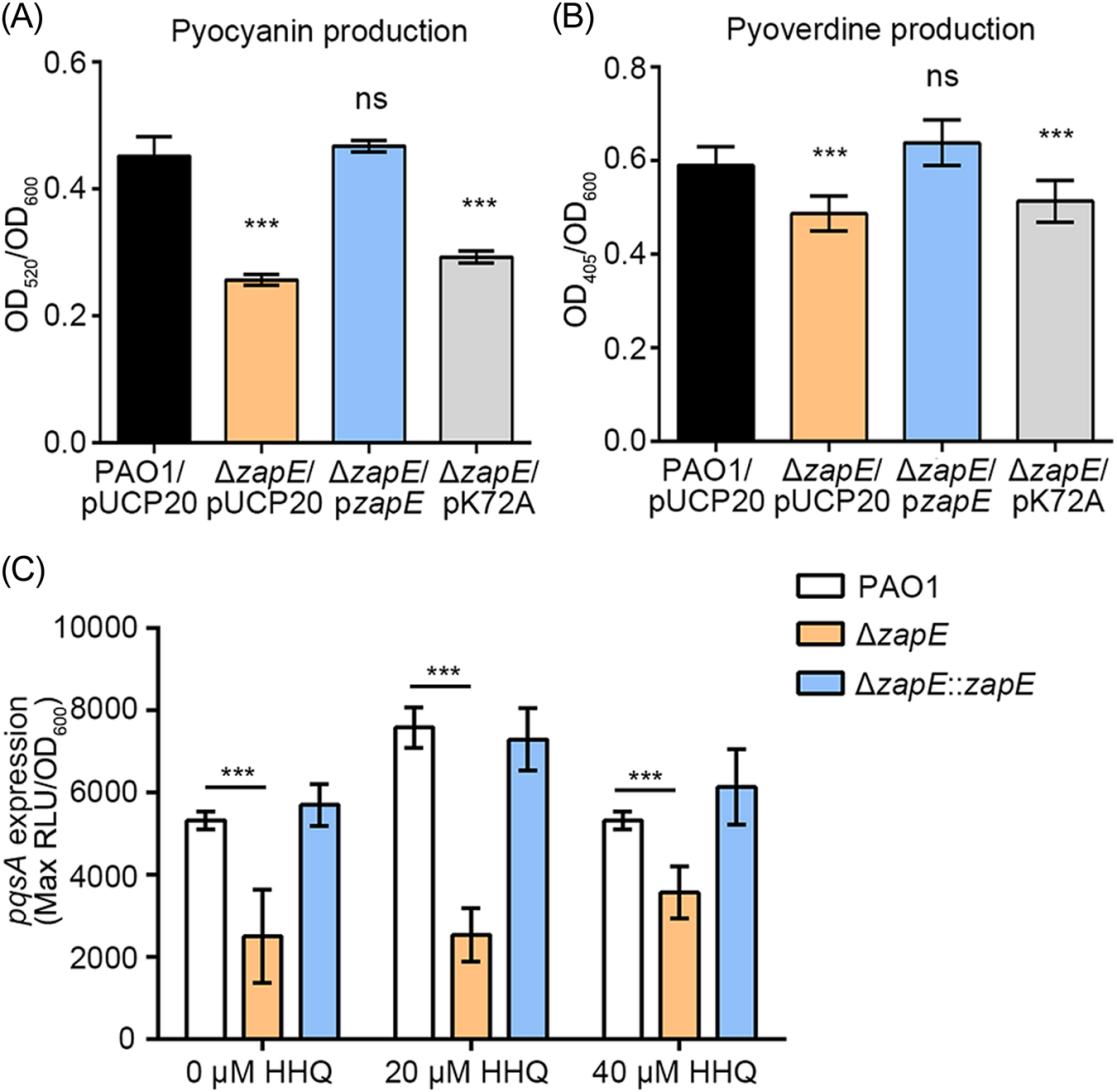
ZapE enhances the synthesis of PQS, pyocyanin, and pyoverdine. (A) Production of pyocyanin of PAO1/pUCP20, Δ*zapE*/pUCP20, Δ*zapE*/p*zapE*, and Δ*zapE*/pK72A. (B) Production of pyoverdine of PAO1/pUCP20, Δ*zapE*/pUCP20, Δ*zapE*/p*zapE*, and Δ*zapE*/pK72A. (C) Maximum *pqsA* expression of PAO1, Δ*zapE*, and Δ*zapE*::*zapE* at different concentrations of HHQ. HHQ was exogenously added into cultures. ***p < 0.001. HHQ, 2‐heptyl‐4‐quinolone; ns, no significant.

Since the *pqs* QS system is involved in autolysis and the release of extracellular DNA, it is activated within a narrow window during the planktonic growth^20^; we thus examined the activation of *pqs* QS in Δ*zapE* and PAO1 by introducing the bioreporter P_*pqsA*_‐*gfp* fusion^20^. The results showed that P_*pqsA*_‐*gfp* expression was significantly decreased in Δ*zapE* during the entire exponential phase (Figure S6A). We also quantified the production of pyocyanin (a derivative of phenazine) and pyoverdine by PAO1, Δ*zapE*, and the complementation strain, and the results were consistent with the RNA‐seq analysis, the production of pyocyanin and pyoverdine were decreased in the absence of *zapE* (Figures 4B and 5A,B).

Pyoverdine is a siderophore and signal molecule that regulates biofilm formation of *P. aeruginosa* ^21^, ^22^. As the expression of *fpvA* and *fpvB*, which are essential for the uptake of ferric–pyoverdine complexes by *P. aeruginosa* ^23^, ^24^, was also downregulated in Δ*zapE* compared to PAO1 (Table S2), we suspected that iron limitation is a factor causing biofilm reduction of the Δ*zapE* mutant. To test this hypothesis, we added an iron chelator (Dipy, 20 μg/ml) to the biofilm culture to cause an iron‐limited environment. PAO1 formed less robust biofilm structures with smaller colonies in the iron‐limiting medium compared to the normal growth medium, which was similar to the biofilm morphology of Δ*zapE* grown in the normal growth medium (Figure S7A). As expected, Δ*zapE* could rarely form regular biofilm structures in iron‐limiting conditions (Figure S7A). To further confirm the effect of iron on the Δ*zapE* mutant biofilm formation, ferric iron was exogenously added into the cultures of PAO1 and Δ*zapE*. The results showed that iron promoted the biofilm formation of the Δ*zapE*, although the overall amount of biomass did not reach the level obtained by PAO1 (Figure S7B).

### ZapE interacts with PqsH

To further elucidate the link between ZapE and *pqs* QS, immunoprecipitation and mass spectrometry (IP‐MS) were used to identify potential ZapE‐interacting proteins. Remarkably, four proteins with well‐known functions in relation to PQS and phenazine synthesis, including PqsH, PhzD1, PhzD2, and PhzM, were identified as potential interacting proteins of ZapE, besides the only known interacting target FtsZ^19^ (Table 2). A surface plasmon resonance (SPR) assay was performed to verify the interaction between ZapE and PqsH, and the results suggested that ZapE could directly interact with PqsH (Figure S6B).

**Table 2:** List of identified ZapE‐interacting proteins related to cell division, PQS, and phenazine synthesis.

Protein name	Uniport ID	Mascot score*	*E* value	Description
FtsZ	FTSZ_PSEAE	158.14	8.0E−06	Cell division protein
PqsH	PQSH_PSEAE	142.81	1.40E−07	Probable flavin adenine dinucleotide (FAD)‐dependent monooxygenase
PhzD1	PHZD1_PSEAE	116.68	6.79E−06	Phenazine biosynthesis protein
PhzD2	PHZD2_PSEAE	116.68	6.79E−06	Phenazine biosynthesis protein
PhzM	PHZM_PSEAE	109.9	2.99E−06	Phenazine‐1‐carboxylate *N*‐methyltransferase

* Mascot score ≥ 100 as threshold.

### ZapE is an ATPase that is required for the synthesis of PQS

PqsH is an NADH‐dependent monooxygenase, which converts HHQ to PQS^13^. As ZapE was shown to interact with PqsH and there was no difference in expression of *pqsH* in Δ*zapE* compared to PAO1 according to RNA‐seq analysis (Figure 4B), we hypothesized that the low level of *pqs* QS of Δ*zapE* might be caused by the reduced enzyme activity of PqsH. To verify this, liquid chromatography‐tandem mass spectrometry (LC‐MS/MS) was used to quantify the amount of HHQ and PQS in PAO1/pUCP20, Δ*zapE*/pUCP20, and Δ*zapE*/p*zapE*. As expected, the results confirmed that Δ*zapE*/pUCP20 produced less PQS compared to the wild‐type PAO1/pUCP20 (Figure S6C), while there was no statistical difference in HHQ production between these three strains (Figure S6D). To further test PqsH enzyme activity, HHQ was exogenously added into cultures of PAO1 and Δ*zapE*, and the PQS bioreporter *pqsA‐gfp* was used to assess the *pqs* QS levels of these two strains. In the absence of *zapE*, exogenous HHQ did not restore *pqs* QS activity to the level of PAO1 (Figure 5C). Furthermore, Δ*zapE* cell lysates with exogenously added recombinant ZapE at 1 μM produced a higher amount of PQS compared to PAO1 cell lysates (Figure S6E), suggesting that ZapE promotes the conversion of HHQ to PQS. To further confirm whether higher amounts of ZapE increase the activity of PqsH in vivo, the *pqs* QS levels of a *zapE* chromosomal complementation strain ∆*zapE*::*zapE* and a *zapE* overexpression strain PAO1::*zapE* were measured. The results showed that the chromosomal complementation strain ∆*zapE*::*zapE* restored *pqs* QS to PAO1 levels; however, the overexpression strain PAO1::*zapE* produced a lower amount of PQS compared to PAO1, suggesting that the higher amounts of ZapE did not increase the activity of PqsH (Figure S6F).

Since ZapE is predicted to be an ATPase belonging to AAA+ proteins, we measured ATP hydrolysis activity of ZapE. The results showed that ZapE is a Ca^2+^/Mg^2+^ ATPase (Figure S6G). An alanine substitution was introduced into the predicted ATP binding site of ZapE (K72A), which decreased the ATPase activity of ZapE (K72A) 10‐fold compared to ZapE (Figure S6H). To explore whether the ATPase activity of ZapE is involved in PQS synthesis and *pqs* QS, we constructed the Δ*zapE*/K72A mutant for the phenotypic assays. The Δ*zapE*/K72A mutant did not produce PQS, pyocyanin, or pyoverdine to levels reached by wild‐type PAO1 (Figures S6C and 5A–C). This suggests that the ATPase enzyme activity of ZapE is required for the synthesis of PQS and the *pqs* QS.

### ZapE is highly conserved among the *Pseudomonas* species

**Figure 6:**
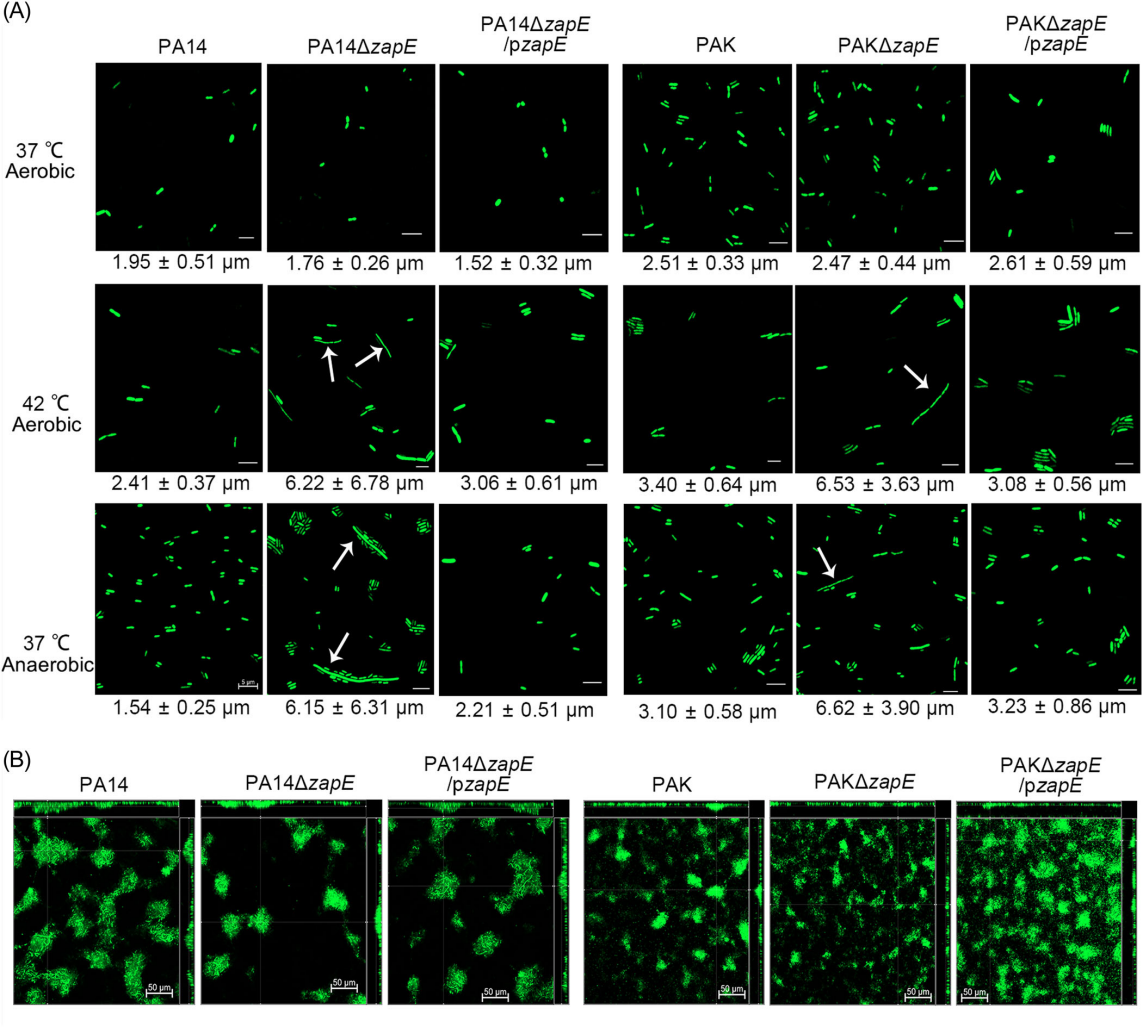
ZapE is required for proper cell division and biofilm formation of PA14 and PAK. (A) Morphology of PA14, PA14Δ*zapE*, PA14Δ*zapE*/p*zapE*, PAK, PAKΔ*zapE*, and PAKΔ*zapE*/p*zapE* in 37°C aerobic, 42°C aerobic, and 37°C anaerobic environment. The white arrows point to the elongated cells. Scale bar = 5 μm. (B) Biofilm morphology of PA14, PA14Δ*zapE*, PA14Δ*zapE*/p*zapE*, PAK, PAKΔ*zapE*, and PAKΔ*zapE*/p*zapE* under 37°C aerobic condition. The biofilm was stained by SYTO9. Scale bar = 50 μm.

The amino acid sequence of ZapE appeared to be highly conserved not only in *P. aeruginosa* strains (PAO1, PA14, and PAK), but also in other members of the *Pseudomonas* group, such as *Pseudomonas stutzeri*, *Pseudomonas putida*, *Pseudomonas chloritidismutans*, and *Pseudomonas fluorescens* (Figure S3). To examine whether ZapE plays a key role in the biofilm formation and cell division in other *P. aeruginosa* strains, *zapE* in‐frame deletion mutants were constructed in PA14 and PAK. The cell shape and biofilm morphology of *zapE* mutants (PA14Δ*zapE* and PAKΔ*zapE*), wild‐type strains (PA14 and PAK), and their complementation strains (PA14Δ*zapE*/p*zapE* and PAKΔ*zapE*/p*zapE*) were observed by CLSM. Both PA14Δ*zapE* and PAKΔ*zapE* had elongated cells under anaerobic and high‐temperature conditions (Figure 6A). And the cell size of their complementation strains restored to the level of their wild type. More importantly, biofilms formed by PA14Δ*zapE* and PAKΔ*zapE* had thinner layers and were less aggregated in comparison to their wild‐type strains and complementation strains (Figure 6B).

## DISCUSSION

*P. aeruginosa* is a leading nosocomial human pathogen with the capability to form biofilms. Its biofilm mode of growth is associated with chronic infections, which are difficult to eradicate due to their resilience to antibiotic therapy and host immune clearance^25^. Currently, approaches to understand biofilm formation mechanisms rely on the characterization of single gene knockout mutants of the model strains, which may not resemble natural conditions. In natural ecological systems, bacteria are nested in communities with highly intraspecific diversity, where variants with different biofilm formation capacities evolve. In this study, we applied the high‐throughput Tn‐seq method in combination with the robust tubular biofilm growth system to mimic the clinical biofilms where a considerable number of variants coexist and compete with each other. This integrated approach enables us to identify new genes that are vital for *P. aeruginosa* biofilm formation and maintenance, some of which have no predicted function (Figure 1E).

In this study, we characterized a new protein involved in biofilm development, ZapE (PA4438), which was shown to be a cell division factor and a Ca^2+^/Mg^2+^ ATPase belonging to the AFG1 (ATPase family gene 1) AAA+ proteins^26^. The AAA+ (ATPases associated with diverse cellular activities) proteins can generate energy from adenosine triphosphate (ATP) hydrolysis and are known to play significant roles in many biochemical processes, such as gene expression, chaperoning protein folding or unfolding^27^. AAA+ proteins are recognized by their Walker A and Walker B motifs and an extra β‐strand between these two motifs^26^, ^27^, ^28^, ^29^. So far, little is known about whether AAA+ proteins are involved in bacterial biofilm formation.

As ZapE is essential for bacterial cells to survive and maintain proper cell shape under anaerobic conditions (Figure 3), we initially speculated that the reduced biofilm biomass and loss of fitness of the Δ*zapE* mutant compared to the wild‐type PAO1 strain were due to the lack of oxygen as a thick biofilm developed. However, we noticed that the Δ*zapE* mutant lost fitness against the PAO1 strain in static biofilm cocultures where only thin‐layered biofilms developed. RNA‐seq analysis revealed that the Δ*zapE* mutant turned down the expression of most *pqs* QS regulated genes, which are critical for *P. aeruginosa* biofilm formation. The *pqs* QS regulates *P. aeruginosa* via several different mechanisms. First, *pqs* QS regulates the release of extracellular DNA and membrane vesicles, which serve as EPS components of *P. aeruginosa* biofilms^30^, ^31^. Second, *pqs* QS regulates the synthesis of pyocyanin, which not only promotes the release of extracellular DNA^32^, but also facilitates the binding of extracellular DNA to the cell surface of *P. aeruginosa* ^33^. Third, *pqs* QS positively regulates the synthesis of pyoverdine^34^, known to enhance subpopulation interactions and formation of large microcolonies of *P. aeruginosa* biofilms^35^. PQS is also an iron chelator, which helps *P. aeruginosa* to overcome iron limitation during infections^34^. Several efficient *P. aeruginosa* biofilm‐inhibiting compounds have been developed by targeting *pqs* QS^36^, ^37^. Recent studies have revealed the immune‐modulating functions of the PQS molecules, which again might enhance the progress of *P. aeruginosa* infections^38^. Downregulation of *pqs* QS genes and decreased production of *pqs*‐associated compounds for biofilm formation in the ZapE mutant suggest that ZapE regulates biofilm formation by involvement with *pqs* QS networks.

**Figure 7:**
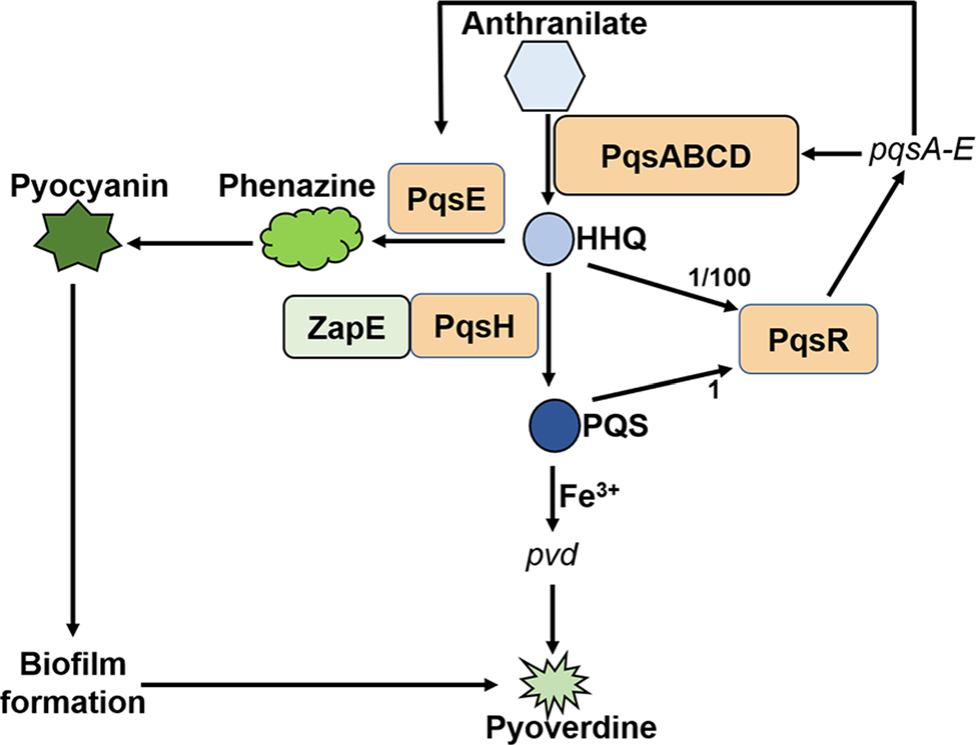
Model of the ZapE regulation in the *pqs* quorum sensing system in *P. aeruginosa*. HHQ is synthesized by PqsABCD and converted to PQS by PqsH. ZapE is a cell division factor, which can directly interact with PqsH and positively regulate the synthesis of PQS. Both HHQ and PQS can bind to the transcriptional regulator PqsR; however, the affinity of PQS is approximately 100‐fold more potent than HHQ at stimulating PqsR activity. Autoinduction occurs when either HHQ or PQS binds to PqsR, and then the expression of *pqsA‐E* operon is activated. PqsE is a putative metallohydrolase protein, which positively regulates pyocyanin biosynthesis as well as biofilm formation. The biosynthesis of pyoverdine can be positively impacted by the biofilm and the iron chelator PQS. In brief, ZapE regulates *P. aeruginosa* biofilm formation by impacting the *pqs* quorum sensing.

We identified that PqsH is a target protein of ZapE (Table 2 and Figure S6B), which provides a direct link between ZapE and *pqs* QS. To explore the binding interactions of PqsH with ZapE, protein–protein docking simulations were carried out. Since the three‐dimensional (3D) structure of ZapE is unknown, we constructed its 3D structure by AlphaFold (Figure S8A,B), which is a novel machine learning approach that incorporates physical and biological knowledge about protein structure^39^. The results suggested that the PqsH residues Arg168, Arg332, Lys333, Trp336, Arg340, Ser364, Arg367, and Arg371 are involved in binding with the ZapE residues Pro14, Phe16, Asp19, Ser197, Gly18, Asp200, Arg204, Gln208, Glu351, Gln353, Glu356, and Thr359 through salt bridges and hydrogen bond interactions (Figure S8C,D and Table S3). PqsH is an NADH‐dependent flavin monooxygenase that oxidizes HHQ to PQS^13^. Our results showed that exogenous HHQ could not restore the *pqs* QS of Δ*zapE* to PAO1 levels. The *zapE* chromosomal complementation strain Δ*zapE*::*zapE* (Figure 5D) and Δ*zapE* cell lysates with exogenously added recombinant ZapE at 1 μM (Figure S6E) had higher *pqs* QS levels than Δ*zapE*. We also showed that complementation of the Δ*zapE* mutant using an ATPase activity‐deficient K72A ZapE variant failed to restore *pqs* QS to PAO1 levels. Our work provides strong evidence for the involvement of the ATPase activity of ZapE in the synthesis of *pqs* QS molecules (Figure 7).

In summary, our work showed that ZapE, a cell division factor and an AAA+ protein, is a novel biofilm regulator through modulating the *pqs* QS. To our knowledge, this is the first report of an AAA+ protein as a biofilm determinant and *pqs* QS regulator. As the ZapE amino acid sequence is highly conserved among the *Pseudomonas* group, this study could also provide a novel target for the development of anti‐*Pseudomonas* compounds.

## MATERIALS AND METHODS

### Bacterial strains and growth conditions

The bacterial strains and plasmids used in this study are described in supplementary Table S4. Unless otherwise indicated, all *P. aeruginosa* strains were isogenic of wild‐type strain PAO1 and grown at 37°C in Luria broth (LB; Sangon), 1/10 LB (vol/vol) or ABTGC media, a chemically defined media^35^. *E. coli* strains were grown at 37°C in LB. Antibiotics were added to the appropriate media at the following concentrations: for *P. aeruginosa*, gentamycin (Gm; Sangon) 30 µg/ml, carbenicillin (Carb, Sangon) 200 µg/ml; for *E. coli*, Gm 30 µg/ml, ampicillin (Ap; Sangon) 100 µg/ml, chloramphenicol (Cm, Sangon) 6 µg/ml, kanamycin (Kan; Sangon) 50 µg/ml. l‐Arabinose (MilliporeSigma) was added into the medium when strains contain the l‐arabinose inducible promoter P_BAD_.

### Transposon insertion mutant library generation

The pBT20 was chosen to construct the mutant library in this study, following the standardized method reported previously^40^. In brief, triparental conjugation was used to introduce pBT20 into *P. aeruginosa* PAO1. The transposon mutants were harvested and mixed thoroughly, which were adjusted to a bacterial density at OD_600_ ~ 2. The transposon mutant suspension was preserved in 50% glycerol at −80°C with 1 ml per cryotube. The transposon insertion was examined by arbitrary PCR using primers Pa‐Rnd1‐F and BT20‐Rnd1‐R, Pa‐Rnd2‐F, and BT20‐Rnd2‐R (Table S5).

### Biofilm cultivation of Tn‐seq mutant libraries

Tubing‐biofilms of the PAO1‐Tn insertion mutant library were cultivated at 37°C following the standard method described previously^41^, ^42^. In brief, the preculture for biofilm inoculation was prepared by enrichment of triplicate, parallel PAO1‐Tn mutant library stocks in LB medium (1:9 vol/vol) for 6 h. The precultures were mixed thoroughly followed by adjusting the cell concentration to OD_600_ = 1. Thereafter, 1 ml of mixed preculture (input) was inoculated into replicate tubular biofilm systems and allowed initial attachment of bacteria for 2 h before starting the flow. The feeding medium was 1/10 LB medium, and the flow rate was set at 4 ml/h. Biofilms in the tubular systems were harvested after 3 days of cultivation (output) for genome DNA extraction.

### Sequencing and analysis of transposon mutant libraries

The genomic DNA of both input and output transposon mutant libraries were extracted by the QIAamp DNA Mini Kit (Qiagen). The transposon sequencing library was prepared using a modified method reported previously^43^. Genomic DNA was sheared to 200–500 bp fragments, before end repair and dA‐tailing using the TIANSeq fragmentation/end repair/dA‐tailing module (Tiangen). Illumina multiplexing adaptors (Tn‐AD‐1 and Tn‐AD‐2) were ligated onto the repaired genomic DNA by the TIANSeq Fast Ligation module (Tiangen). Thereafter, transposon junction sites were amplified with primers (Tn‐Rnd1‐F and Tn‐Rnd1‐R, Tn‐Rnd2‐F, and Tn‐Rnd2‐R), which recognize the end of the transposon and adaptor by Phusion Hot Start II High‐Fidelity PCR Master Mix (Thermofisher). All adaptors and primers used in this study are listed in Table S5. The raw data of transposon mutant library sequencing were trimmed and mapped to the PAO1 reference genome. Unique gene reads were recorded and normalized based on the DESeq2 normalization method. Statistical analysis was performed by Origin9 (OriginLab Corporation), GraphpadPrism9 (Graphpad), and R‐packages (https://www.r-project.org/). Significance was determined by criteria: (a) log_2_|Fold‐change| > 2; (b) *p* value via multiple *t* test < 0.05.

### In‐frame deletion mutants, site‐directed mutants, and complementation strain construction

To construct *P. aeruginosa* mutants, pK18‐Gm‐mobsacB was used as the vector of the knockout plasmid^44^, and all primers used in this study are listed in Table S5. First, upstream and downstream fragments of the target gene were amplified (Uprimer‐F and Uprimer‐R, Dprimer‐F, and Dprimer‐R) and fused into the knockout plasmid. Then, the target gene deletion mutant was constructed by in‐frame deletion^45^. The complementation strain was constructed using the complementation plasmid pUCP20. The target gene was amplified and fused into pUCP20, which was transformed into the target gene deletion mutant by electroporation. To construct the ZapE_K72A_ site‐directed mutant, the complementation plasmid pUCP20‐*zapE* was used as the template, and K72A‐F and K72A‐R were applied as PCR primers. To construct the *zapE* chromosomal complementation strain, an integration plasmid miniCTX‐*lacZ* was used. The promoter region and the open reading frame of *zapE* were cloned into miniCTX‐*lacZ*, and CTX‐*zapE*‐F and CTX‐*zapE*‐R were applied as PCR primers. The resultant plasmid was transformed into ∆*zapE* and PAO1 to construct the *zapE* chromosomal complementation strain ∆*zapE*::*zapE* and *zapE* overexpression strain PAO1::*zapE*, respectively. Reagents and kits used in this section included the QIAamp DNA Mini Kit (Qiagen), HiPure Gel Pure DNA Mini Kit (Magen), HiPure Plasmid Micro Kit (Magen), Q5 High‐Fidelity 2× Master Mix (NEB), PrimeSTAR® HS DNA Polymerase with GC Buffer (Takara), and Gibson Assembly Master Mix (NEB).

### Transcriptomic sequencing analysis

The total RNA was extracted with a QIAmp RNA kit from liquid culture of PAO1 and its mutant strain at the late exponential growth stage. The mRNA was purified using the NEBNext Poly(A) mRNA Magnetic Isolation Module (NEB), and the sequencing library was prepared using the NEBNext Ultra II mRNA Library Prep Kit for Illumina (NEB). The library quality was examined by Qubit dsDNA high‐sensitivity assay (Thermofisher) and D1000 Screen Tape (Agilent) for concentration and fragmentation. Transcriptomic sequencing was performed on NovaSeq. 6000 (Illumina Inc.) with a NovaSeq S4 reagent kit. The transcriptomic raw data were mapped to the PAO1 reference genome by the CLC genomics workbench (CLC bio, Qiagen) and were analyzed by DESeq statistical analysis. Significance was determined by criteria: (a) log_2_|Fold‐change| > 2; (b) *p* value via multiple *t* test < 0.05.

### Biofilm assay and imaging analysis

Biofilm formation and structures were evaluated by CLSM (Zeiss LSM 900). Static biofilms were prepared by inoculating overnight bacterial cultures at a final concentration of OD_600_ ~ 0.01 and were cultivated for 24 h at 37°C. Competition biofilm assay was prepared by mixing PAO1 with PAO1 and its mutant at 1:1 vol:vol with final bacterial concentration at OD_600_ ~ 0.01, followed by cultivation. CLSM 3D images were captured by LSM900 and analyzed by Imaris (Imaris, bitplane), ImageJ (https://imagej.nih.gov/ij/), and Zen (Zeiss).

### Evaluation of growth and cell size of bacteria

The growth of PAO1 and its mutant as well as complementation strains were continuously monitored by a microplate reader (Tecan Spark). Overnight strain cultures were adjusted to a concentration of OD_600_ ~ 0.01 in LB broth, which were allowed to grow at 37°C for 16 h with periodic shaking. Growth was monitored by measuring absorbance at 600 nm every 15 min. Bacterial cell sizes under four different cultivation conditions were evaluated using CLSM imaging. The four cultivation conditions include: (I) liquid culture in a 37°C shaker; (II) liquid culture in a 42°C shaker; (III) liquid culture in an anaerobic bag in a 37°C incubator; and (IV) liquid culture in an aerobic 37°C incubator. ImageJ (https://imagej.nih.gov/ij/) was used to measure the cell size of bacteria from CLSM images.

### Motility assays

Motility assays were carried out as previously described with minor modifications^46^, ^47^. Twitching motility was conducted by stabbing an isolate to the bottom of the LB plate (1.2% agar). Swimming motility was performed by stabbing an isolate on the surface of the LB plate (0.3% agar). After 24 h of incubation at 37°C, motility zones were measured. At least three replicates were measured for each sample.

### Congo red assay

To assay colony morphology, Congo red plates (10 g/L tryptone, 150 μg/ml Congo red, 1% agar) were used. Overnight strain cultures were adjusted to a concentration of OD_600_ ~ 0.01 in PBS (phosphate‐buffered saline) buffer, and 5 μL of the culture was pipetted on plates. The plates were incubated at room temperature.

### Bioreporter quantification of c‐di‐GMP and PQS

The c‐di‐GMP and PQS levels in PAO1 wild‐type and its mutants were measured by the fluorescent intensity of a green‐fluorescent‐protein tagged reporter P*cdrA*‐*gfp* ^48^ and P*pqsA*‐*gfp* ^49^, respectively. The fluorescent intensity was recorded by a Tecan Spark microplate reader with an excitation wavelength of 485 nm and an emission wavelength of 535 nm.

### Pyoverdine and pyocyanin assays

PAO1, its mutants, and complementation strains were incubated for 24 h in LB broth. The pyoverdine production was quantified by reading the absorbance of the supernatant at 405 nm and then normalized by OD_600_ of the culture (Tecan Spark)^34^, ^50^. For phenazine pigment–pyocyanin assay, cultures were adjusted to a concentration of OD_600_ ~ 0.1 in PBS buffer, and then, 100 μL of culture was spread on PIA plates (Pseudomonas Isolation Agar; BD Biosciences). Pyocyanin was extracted with chloroform and 0.5 M HCl and the absorbance was measured at 520 nm (Tecan Spark) as previously described^51^.

### LC‐MS/MS analysis of HHQ and PQS

One milliliter of culture fluid was centrifuged at 12,000 rpm for 2 min; the supernatant was filtered through a 0.2 μm Millex Syring Filter and then mixed with an equal volume of methanol. The crude extract was subjected to LC‐MS/MS as previously reported^52^, ^53^. All MS experiments in this study were performed on an AB SCIEX QTRAP 4500 (Applied Biosystem). Analyst software was applied for data acquisition and processing.

### Protein expression and purification

To express ZapE and K72A, the expression vector pET28a was used. The *zapE* and *zapE*_*K72A*_ each were amplified from PAO1 genomic DNA and pUCP20‐K72A with 28a‐*zapE*‐F and 28a‐*zapE*‐R. For PqsH, the expression vector pMAL‐C5x was chosen, and the *pqsH* was amplified with MAL‐*pqsH*‐F and MAL‐*pqsH*‐R. Gibson Assembly Master Mix (NEB) was applied to fuse the amplicons with the expression vectors. The fusion gene constructs were then transformed into *E. coli* strain BL21 (DE3). HIS‐ZapE and HIS‐K72A were purified on Ni NTA beads (Smart Lifesciences) independently^19^, and MBP‐PqsH was purified on Dextrin Beads 6FF (Smart Lifesciences) as previously reported^13^. The fusion proteins were further purified by ion exchange on a Mono Q 5/50 GL column (GE Biosciences) and then followed by a gel filtration on a HiLoad 16/60 Superdex 200 size exclusion column (GE Biosciences).

### SPR analysis

The PqsH protein in 20 mM HEPES (pH 8.0) and 150 mM NaCl (HEPES buffered Steinberg's Solution, HBST buffer) was fixed on a CM 5 chip, and then, ZapE protein was serially diluted in HBST buffer. The binding affinities of PqsH and ZapE were measured by the single cycle mode of BIAcore T200 at 25°C. Biacore T200 software version 3.0 with a 1:1 Langmuir binding model was applied to analyze the binding kinetics of proteins.

### IP‐MS

Genomic loci of *zapE* without the stop codon and *gfp* were cloned into pHerd20T, and the gene locus of *gfp* was cloned into pHerd20T as a control. All primers used in this section are listed in Table S5. The resulting vectors p20T‐*zapE*‐*gfp* and p20T‐*gfp* were transformed into the Δ*zapE* separately. The total proteins were incubated with GFP‐trap Agarose (Chromotek) as previously described^54^. LC‐MS/MS analysis was carried out by Wininnovate Bio Co. Ltd.

### ATPase activity assay

A ultramicro ATPase activity assay kit for Na^+^/K^+^ ATPase, Ca^2+^/Mg^2+^, and all types of ATPase (Nanjing Jiancheng) was used to test the activity of the purified ZapE and K72A in this study. The ATPase activity of samples is calculated by the formula *B*/(*t* × *V*) × *D* (*B* is the phosphate amount, *t* is the reaction time, *V* is the sample volume added into the reaction well, and *D* is the sample dilution factor).

### Protein–protein docking

The 3D structure of ZapE was constructed by AlphaFold^39^. The protein–protein docking module in ClusPro^55^ was used to predict the molecular docking of ZapE and PqsH (ID: AF‐Q9I0Q0‐F1‐model_v2, AlphaFold Protein Structure Database) as previously reported^56^. Molecular operating environment (MOE) was chosen to predict the contacting residues of interacting proteins.

### Statistical analysis

The results in this article are presented as the mean ± SD of at least three independent replicates. Student's unpaired *t* test was used to evaluate significance. ***p < 0.001; **p < 0.01; *p < 0.05; ns, no significant.

## AUTHOR CONTRIBUTIONS

Liang Yang and Lianhui Zhang conceived the project. Xi Liu, Minlu Jia, Yingdan Zhang, Jing Wang, Zhaoxiao Yu performed experiments. Xi Liu, Yingdan Zhang, Yang Liu, Hang Cheng, Zhao Cai and Luyan Z. Ma analyzed data. Xi Liu, Yingdan Zhang, Liang Yang and Lianhui Zhang wrote the manuscript.

## ETHICS STATEMENT

There is no animal used in this study.

## CONFLICT OF INTERESTS

The authors declare no conflict of interests.

## Supporting information

Supporting information.

## Data Availability

The Illumina sequencing transcriptomic data used in this study could be found in BioProject (No. PRJNA701135) on the NCBI database. The data that support the findings of this study are available from the corresponding authors upon reasonable request.
