# Supplementary material for: Cell division factor ZapE regulates Pseudomonas aeruginosa biofilm formation by impacting the pqs quorum sensing system
Source: mLife. 2023 Mar 21;2(1):28–42. doi: 10.1002/mlf2.12059 (PMC10989928; doi:10.1002/mlf2.12059)
Supplement: Supplementary file 1 — Supporting information. [file MLF2-2-28-s001.docx]

**The cell division factor ZapE regulates *Pseudomonas aeruginosa* biofilm formation by impacting the *pqs* quorum sensing system**

Xi Liu^1,2,3^, Minlu Jia^2^, Jing Wang^2^, Hang Cheng^2^, Zhao Cai^2^, Zhaoxiao Yu^1^, Yang Liu^4^, Luyan Z Ma^1^, Lianhui Zhang^3,*^, Yingdan Zhang^2,*^, Liang Yang^2,5*^

^1^ State Key Laboratory of Microbial Resources, Institute of Microbiology, Chinese Academy of Sciences, Beijing, China

^2^ School of Medicine, Key University Laboratory of Metabolism and Health of Guangdong, Southern University of Science and Technology, Shenzhen, China

^3^ Guangdong Province Key Laboratory of Microbial Signals and Disease Control, Integrative Microbiology Research Center, South China Agricultural University, Guangzhou, China

^4^ Medical Research Center, Southern University of Science and Technology Hospital, Shenzhen, China

^5^ Shenzhen Third People’s Hospital, The Second Affiliated Hospital of Southern University of Science and Technology, National Clinical Research Center for Infectious Disease, Shenzhen 518112, China

**^*^ Corresponding authors**

Email: [yangl@sustech.edu.cn](mailto:yangl@sustech.edu.cn), zhangyd6@sustech.edu.cn or [lhzhang01@scau.edu.cn](mailto:lhzhang01@scau.edu.cn)

**Short title**: Identification of genetic determinants for *P. aeruginosa* biofilm via Tn-seq

**Table S1. Significant genes identified in output samples.**

| **Gene** | **PA** | **Output/Input** | **p-value** |
| --- | --- | --- | --- |
| aceF | PA5016 | 0.21 | 0.02 |
| aceK | PA1376 | 0.17 | 0.00 |
| acsA | PA0887 | 0.22 | 0.02 |
| algA | PA3551 | 0.23 | 0.02 |
| algC | PA5322 | 0.12 | 0.00 |
| algD | PA3540 | 0.12 | 0.01 |
| algF | PA3550 | 0.11 | 0.00 |
| algJ | PA3549 | 0.19 | 0.00 |
| algR | PA5261 | 0.14 | 0.01 |
| algX | PA3546 | 0.19 | 0.00 |
| algZ | PA5262 | 0.08 | 0.00 |
| alkB2 | PA1525 | 0.16 | 0.01 |
| ambA | PA2306 | 0.13 | 0.00 |
| amiC | PA3364 | 0.17 | 0.05 |
| amiE | PA3366 | 0.20 | 0.01 |
| amn | PA3970 | 0.16 | 0.00 |
| ampDh3 | PA0807 | 0.10 | 0.01 |
| ampG | PA4393 | 0.22 | 0.01 |
| ansA | PA2253 | 4.50 | 0.05 |
| aotJ | PA0888 | 0.16 | 0.00 |
| apaH | PA0590 | 0.04 | 0.00 |
| aprA | PA1249 | 0.18 | 0.01 |
| aprE | PA1247 | 0.15 | 0.01 |
| aprF | PA1248 | 0.17 | 0.03 |
| argF | PA3537 | 0.22 | 0.00 |
| argG | PA3525 | 0.16 | 0.01 |
| arnA | PA3554 | 0.25 | 0.02 |
| aroP1 | PA3000 | 0.15 | 0.01 |
| aruI | PA4977 | 0.23 | 0.00 |
| atuA | PA2886 | 0.24 | 0.00 |
| atuR | PA2885 | 0.12 | 0.01 |
| betA | PA5372 | 0.17 | 0.00 |
| betC | PA0031 | 0.13 | 0.01 |
| betT1 | PA5375 | 0.14 | 0.04 |
| bfmS | PA4102 | 0.10 | 0.02 |
| braD | PA1073 | 0.18 | 0.03 |
| braZ | PA1971 | 0.23 | 0.00 |
| cafA | PA4477 | 0.11 | 0.00 |
| ccoN2 | PA1557 | 0.06 | 0.00 |
| ccoO1 | PA1553 | 0.22 | 0.05 |
| ccoP2 | PA1555 | 0.09 | 0.00 |
| cdhB | PA5385 | 0.08 | 0.01 |
| cdhR | PA5389 | 0.25 | 0.01 |
| chpA | PA0413 | 0.07 | 0.01 |
| coaD | PA0363 | 0.19 | 0.03 |
| cobB | PA1273 | 0.25 | 0.01 |
| cobM | PA2948 | 0.17 | 0.00 |
| codB | PA0438 | 0.18 | 0.02 |
| corA | PA5268 | 0.21 | 0.00 |
| coxA | PA0106 | 0.19 | 0.02 |
| cpo | PA2717 | 0.08 | 0.01 |
| crc | PA5332 | 0.06 | 0.01 |
| cupB2 | PA4085 | 0.21 | 0.03 |
| cupB3 | PA4084 | 0.16 | 0.00 |
| cupC1 | PA0992 | 0.07 | 0.00 |
| cupC3 | PA0994 | 0.22 | 0.01 |
| cyaA | PA5272 | 0.14 | 0.00 |
| cyaB | PA3217 | 0.08 | 0.00 |
| cyoA | PA1317 | 0.19 | 0.02 |
| cyoB | PA1318 | 0.21 | 0.01 |
| cyoE | PA1321 | 0.17 | 0.04 |
| cysB | PA1754 | 0.04 | 0.00 |
| cysP | PA1493 | 0.20 | 0.01 |
| cysT | PA0282 | 0.22 | 0.03 |
| cysW | PA0281 | 0.16 | 0.00 |
| dauA | PA3863 | 0.19 | 0.01 |
| dctA | PA1183 | 0.20 | 0.03 |
| ddlA | PA4201 | 0.19 | 0.00 |
| dgcA | PA5398 | 0.16 | 0.00 |
| dht | PA0441 | 0.16 | 0.00 |
| dipZ | PA4845 | 0.16 | 0.01 |
| dnr | PA0527 | 0.14 | 0.00 |
| dsbC | PA3737 | 0.12 | 0.00 |
| dsbH | PA5256 | 0.15 | 0.03 |
| ercS | PA1992 | 0.23 | 0.00 |
| exbB1 | PA0198 | 0.14 | 0.00 |
| exoT | PA0044 | 0.21 | 0.00 |
| exsA | PA1713 | 0.10 | 0.01 |
| fdhE | PA4809 | 0.12 | 0.01 |
| fepC | PA4158 | 0.18 | 0.00 |
| fgtA | PA1091 | 0.19 | 0.05 |
| fha1 | PA0081 | 0.21 | 0.00 |
| fiuA | PA0470 | 0.18 | 0.00 |
| fleN | PA1454 | 0.09 | 0.01 |
| fliM | PA1443 | 0.25 | 0.03 |
| foxR | PA2467 | 0.19 | 0.00 |
| fpvB | PA4168 | 0.17 | 0.00 |
| fpvI | PA2387 | 0.10 | 0.01 |
| fumC1 | PA4470 | 0.13 | 0.00 |
| gabP | PA0129 | 0.12 | 0.00 |
| gacS | PA0928 | 0.12 | 0.00 |
| galE | PA1384 | 0.14 | 0.00 |
| gbt | PA3082 | 0.22 | 0.00 |
| gbuA | PA1421 | 0.24 | 0.03 |
| gcdH | PA0447 | 0.17 | 0.00 |
| gcl | PA1502 | 0.19 | 0.02 |
| gcvP1 | PA5213 | 0.19 | 0.00 |
| gfnR | PA3630 | 0.22 | 0.00 |
| gidA | PA5565 | 0.16 | 0.01 |
| glgP | PA2144 | 0.21 | 0.03 |
| glpF | PA3581 | 0.17 | 0.01 |
| gltS | PA3176 | 0.17 | 0.01 |
| gmd | PA5453 | 0.13 | 0.01 |
| hcp1 | PA0085 | 0.09 | 0.01 |
| hemN | PA1546 | 0.13 | 0.01 |
| hemO | PA0672 | 4.37 | 0.01 |
| hflC | PA4941 | 0.09 | 0.00 |
| hisH1 | PA5142 | 0.09 | 0.04 |
| hisM | PA2925 | 0.16 | 0.01 |
| hisP | PA2926 | 0.13 | 0.02 |
| hitA | PA4687 | 0.11 | 0.01 |
| hpcB | PA4124 | 0.16 | 0.00 |
| hpcC | PA4123 | 0.20 | 0.01 |
| hutG | PA5091 | 0.07 | 0.02 |
| icmF1 | PA0077 | 0.21 | 0.03 |
| ilvA1 | PA0331 | 0.22 | 0.02 |
| ilvC | PA4694 | 0.14 | 0.00 |
| katA | PA4236 | 0.21 | 0.05 |
| katN | PA2185 | 0.15 | 0.01 |
| kdpF | PA1632 | 0.12 | 0.02 |
| kup | PA0917 | 0.22 | 0.01 |
| ladS | PA3974 | 0.19 | 0.03 |
| lipA | PA2862 | 0.19 | 0.00 |
| lon | PA1803 | 0.21 | 0.03 |
| lpxO2 | PA0936 | 0.18 | 0.02 |
| lysA | PA5277 | 0.08 | 0.00 |
| mdoH | PA5077 | 0.12 | 0.01 |
| metF | PA0430 | 0.14 | 0.05 |
| metR | PA3587 | 0.16 | 0.00 |
| metY | PA5025 | 0.15 | 0.02 |
| mexB | PA0426 | 0.08 | 0.00 |
| mexC | PA4599 | 0.14 | 0.04 |
| mexD | PA4598 | 0.23 | 0.02 |
| mexH | PA4206 | 0.12 | 0.00 |
| mltB1 | PA4444 | 0.13 | 0.00 |
| mmsA | PA3570 | 0.17 | 0.00 |
| moaB1 | PA3915 | 0.13 | 0.03 |
| modA | PA1863 | 0.24 | 0.02 |
| msuD | PA2356 | 0.15 | 0.02 |
| mtlZ | PA2344 | 0.17 | 0.03 |
| mtr | PA5434 | 0.19 | 0.00 |
| mucB | PA0764 | 0.09 | 0.00 |
| mucC | PA0765 | 0.12 | 0.01 |
| mucD | PA0766 | 0.10 | 0.01 |
| mucK | PA1019 | 0.14 | 0.04 |
| nadA | PA1004 | 0.16 | 0.01 |
| narG | PA3875 | 0.21 | 0.01 |
| narH | PA3874 | 0.22 | 0.00 |
| narK1 | PA3877 | 0.16 | 0.00 |
| nirQ | PA0520 | 0.18 | 0.01 |
| nirS | PA0519 | 0.18 | 0.00 |
| nqrE | PA2995 | 0.19 | 0.01 |
| nuoA | PA2637 | 0.13 | 0.04 |
| nuoE | PA2640 | 0.05 | 0.00 |
| nuoG | PA2642 | 0.20 | 0.05 |
| nuoI | PA2644 | 0.08 | 0.01 |
| opdC | PA0162 | 0.18 | 0.00 |
| opdO | PA2113 | 0.15 | 0.04 |
| opdP | PA4501 | 0.21 | 0.03 |
| oprO | PA3280 | 0.17 | 0.00 |
| oprP | PA3279 | 0.16 | 0.02 |
| PA0030 | PA0030 | 0.22 | 0.01 |
| PA0032a | PA0032a | 0.09 | 0.00 |
| PA0041a | PA0041a | 0.10 | 0.03 |
| PA0042 | PA0042 | 0.13 | 0.02 |
| PA0043 | PA0043 | 0.23 | 0.04 |
| PA0047 | PA0047 | 0.12 | 0.00 |
| PA0048 | PA0048 | 0.24 | 0.02 |
| PA0052 | PA0052 | 0.12 | 0.00 |
| PA0061 | PA0061 | 0.16 | 0.01 |
| PA0065 | PA0065 | 0.15 | 0.02 |
| PA0070 | PA0070 | 0.16 | 0.00 |
| PA0071 | PA0071 | 0.13 | 0.00 |
| PA0084 | PA0084 | 0.23 | 0.03 |
| PA0087 | PA0087 | 0.15 | 0.01 |
| PA0093 | PA0093 | 0.16 | 0.04 |
| PA0099 | PA0099 | 0.18 | 0.00 |
| PA0100 | PA0100 | 0.11 | 0.02 |
| PA0102 | PA0102 | 0.24 | 0.02 |
| PA0109 | PA0109 | 0.20 | 0.03 |
| PA0122 | PA0122 | 0.10 | 0.02 |
| PA0125 | PA0125 | 10.77 | 0.01 |
| PA0130 | PA0130 | 0.16 | 0.01 |
| PA0134 | PA0134 | 0.24 | 0.01 |
| PA0137 | PA0137 | 0.18 | 0.03 |
| PA0144 | PA0144 | 0.23 | 0.01 |
| PA0146 | PA0146 | 0.21 | 0.00 |
| PA0148 | PA0148 | 0.15 | 0.01 |
| PA0158 | PA0158 | 0.20 | 0.00 |
| PA0170 | PA0170 | 0.11 | 0.01 |
| PA0177 | PA0177 | 0.07 | 0.01 |
| PA0178 | PA0178 | 0.12 | 0.01 |
| PA0179 | PA0179 | 0.17 | 0.02 |
| PA0188 | PA0188 | 0.17 | 0.00 |
| PA0189 | PA0189 | 0.15 | 0.01 |
| PA0194 | PA0194 | 0.24 | 0.02 |
| PA0202 | PA0202 | 0.21 | 0.03 |
| PA0215 | PA0215 | 0.16 | 0.00 |
| PA0217 | PA0217 | 0.22 | 0.01 |
| PA0222 | PA0222 | 0.10 | 0.01 |
| PA0224 | PA0224 | 0.06 | 0.03 |
| PA0226 | PA0226 | 0.17 | 0.02 |
| PA0233 | PA0233 | 0.18 | 0.02 |
| PA0234 | PA0234 | 0.14 | 0.02 |
| PA0238 | PA0238 | 0.20 | 0.00 |
| PA0248 | PA0248 | 0.18 | 0.02 |
| PA0254 | PA0254 | 0.18 | 0.00 |
| PA0256 | PA0256 | 0.14 | 0.02 |
| PA0257 | PA0257 | 0.14 | 0.00 |
| PA0261 | PA0261 | 0.12 | 0.00 |
| PA0262 | PA0262 | 0.16 | 0.00 |
| PA0309 | PA0309 | 0.08 | 0.00 |
| PA0310 | PA0310 | 0.10 | 0.04 |
| PA0322 | PA0322 | 0.25 | 0.00 |
| PA0324 | PA0324 | 0.11 | 0.00 |
| PA0345 | PA0345 | 0.22 | 0.04 |
| PA0346 | PA0346 | 0.17 | 0.04 |
| PA0348 | PA0348 | 0.14 | 0.00 |
| PA0366 | PA0366 | 0.13 | 0.03 |
| PA0367 | PA0367 | 0.14 | 0.01 |
| PA0392 | PA0392 | 0.16 | 0.00 |
| PA0394 | PA0394 | 0.22 | 0.00 |
| PA0397 | PA0397 | 0.19 | 0.01 |
| PA0405 | PA0405 | 0.11 | 0.00 |
| PA0431 | PA0431 | 0.11 | 0.00 |
| PA0448 | PA0448 | 0.18 | 0.02 |
| PA0454 | PA0454 | 0.23 | 0.00 |
| PA0456 | PA0456 | 0.05 | 0.00 |
| PA0476 | PA0476 | 0.19 | 0.00 |
| PA0484 | PA0484 | 0.13 | 0.04 |
| PA0486 | PA0486 | 0.16 | 0.00 |
| PA0492 | PA0492 | 0.18 | 0.03 |
| PA0493 | PA0493 | 0.19 | 0.01 |
| PA0497 | PA0497 | 0.17 | 0.00 |
| PA0498 | PA0498 | 0.19 | 0.00 |
| PA0499 | PA0499 | 0.13 | 0.01 |
| PA0503 | PA0503 | 4.07 | 0.03 |
| PA0507 | PA0507 | 0.19 | 0.01 |
| PA0525 | PA0525 | 0.17 | 0.04 |
| PA0530 | PA0530 | 0.24 | 0.02 |
| PA0537 | PA0537 | 0.13 | 0.00 |
| PA0540 | PA0540 | 0.20 | 0.00 |
| PA0543 | PA0543 | 0.18 | 0.01 |
| PA0544 | PA0544 | 0.18 | 0.01 |
| PA0550 | PA0550 | 0.21 | 0.04 |
| PA0561 | PA0561 | 0.17 | 0.00 |
| PA0563 | PA0563 | 0.11 | 0.01 |
| PA0569 | PA0569 | 0.19 | 0.00 |
| PA0574 | PA0574 | 0.11 | 0.01 |
| PA0586 | PA0586 | 0.11 | 0.02 |
| PA0599 | PA0599 | 0.25 | 0.00 |
| PA0603 | PA0603 | 0.10 | 0.00 |
| PA0605 | PA0605 | 0.17 | 0.00 |
| PA0606 | PA0606 | 0.20 | 0.03 |
| PA0616 | PA0616 | 0.14 | 0.01 |
| PA0618 | PA0618 | 0.12 | 0.04 |
| PA0620 | PA0620 | 0.17 | 0.00 |
| PA0621 | PA0621 | 0.11 | 0.03 |
| PA0629 | PA0629 | 0.18 | 0.00 |
| PA0636 | PA0636 | 0.21 | 0.03 |
| PA0643 | PA0643 | 0.17 | 0.01 |
| PA0644 | PA0644 | 0.02 | 0.05 |
| PA0646 | PA0646 | 0.25 | 0.01 |
| PA0657 | PA0657 | 0.14 | 0.00 |
| PA0669 | PA0669 | 0.18 | 0.01 |
| PA0681 | PA0681 | 0.12 | 0.01 |
| PA0684 | PA0684 | 0.14 | 0.02 |
| PA0685 | PA0685 | 0.19 | 0.00 |
| PA0689 | PA0689 | 0.17 | 0.01 |
| PA0690 | PA0690 | 0.22 | 0.00 |
| PA0692 | PA0692 | 0.15 | 0.00 |
| PA0712 | PA0712 | 0.17 | 0.00 |
| PA0715 | PA0715 | 0.10 | 0.00 |
| PA0724 | PA0724 | 0.23 | 0.04 |
| PA0725 | PA0725 | 0.13 | 0.02 |
| PA0729 | PA0729 | 0.17 | 0.01 |
| PA0736 | PA0736 | 0.23 | 0.02 |
| PA0745 | PA0745 | 0.22 | 0.01 |
| PA0749 | PA0749 | 0.26 | 0.02 |
| PA0754 | PA0754 | 0.15 | 0.01 |
| PA0779 | PA0779 | 0.22 | 0.02 |
| PA0800 | PA0800 | 0.01 | 0.01 |
| PA0801 | PA0801 | 0.15 | 0.01 |
| PA0806 | PA0806 | 0.21 | 0.01 |
| PA0810 | PA0810 | 0.22 | 0.01 |
| PA0814 | PA0814 | 0.18 | 0.01 |
| PA0834 | PA0834 | 0.15 | 0.00 |
| PA0839 | PA0839 | 0.22 | 0.03 |
| PA0850 | PA0850 | 0.15 | 0.00 |
| PA0855 | PA0855 | 0.23 | 0.02 |
| PA0864 | PA0864 | 0.13 | 0.01 |
| PA0902 | PA0902 | 0.19 | 0.02 |
| PA0911 | PA0911 | 0.10 | 0.01 |
| PA0924 | PA0924 | 0.20 | 0.00 |
| PA0925 | PA0925 | 0.13 | 0.00 |
| PA0939 | PA0939 | 0.18 | 0.05 |
| PA0959 | PA0959 | 0.18 | 0.04 |
| PA0988 | PA0988 | 0.15 | 0.01 |
| PA1014 | PA1014 | 0.20 | 0.03 |
| PA1015 | PA1015 | 0.14 | 0.03 |
| PA1016 | PA1016 | 0.14 | 0.00 |
| PA1037 | PA1037 | 0.18 | 0.00 |
| PA1040 | PA1040 | 0.12 | 0.00 |
| PA1044 | PA1044 | 0.25 | 0.04 |
| PA1052a | PA1052a | 0.11 | 0.00 |
| PA1056 | PA1056 | 0.16 | 0.00 |
| PA1062 | PA1062 | 0.16 | 0.05 |
| PA1064 | PA1064 | 0.16 | 0.00 |
| PA1065 | PA1065 | 0.18 | 0.04 |
| PA1088 | PA1088 | 0.11 | 0.01 |
| PA1090 | PA1090 | 0.20 | 0.01 |
| PA1106 | PA1106 | 0.14 | 0.00 |
| PA1112 | PA1112 | 0.11 | 0.00 |
| PA1118 | PA1118 | 0.13 | 0.00 |
| PA1127 | PA1127 | 0.14 | 0.04 |
| PA1132 | PA1132 | 0.12 | 0.05 |
| PA1135 | PA1135 | 0.19 | 0.01 |
| PA1158 | PA1158 | 0.24 | 0.00 |
| PA1166 | PA1166 | 0.17 | 0.02 |
| PA1181 | PA1181 | 0.18 | 0.00 |
| PA1188 | PA1188 | 0.20 | 0.00 |
| PA1189 | PA1189 | 0.20 | 0.00 |
| PA1206 | PA1206 | 0.04 | 0.03 |
| PA1208 | PA1208 | 0.19 | 0.04 |
| PA1211 | PA1211 | 0.15 | 0.03 |
| PA1216 | PA1216 | 0.14 | 0.02 |
| PA1221 | PA1221 | 0.25 | 0.03 |
| PA1242 | PA1242 | 0.11 | 0.04 |
| PA1251 | PA1251 | 4.45 | 0.00 |
| PA1255 | PA1255 | 0.16 | 0.01 |
| PA1256 | PA1256 | 0.23 | 0.03 |
| PA1283 | PA1283 | 0.10 | 0.01 |
| PA1289 | PA1289 | 0.21 | 0.00 |
| PA1291 | PA1291 | 0.16 | 0.00 |
| PA1296 | PA1296 | 0.23 | 0.01 |
| PA1307 | PA1307 | 0.23 | 0.01 |
| PA1313 | PA1313 | 0.24 | 0.01 |
| PA1322 | PA1322 | 0.12 | 0.00 |
| PA1324.1 | PA1324.1 | 0.15 | 0.03 |
| PA1332 | PA1332 | 0.17 | 0.00 |
| PA1334 | PA1334 | 0.20 | 0.01 |
| PA1346 | PA1346 | 0.24 | 0.03 |
| PA1347 | PA1347 | 0.17 | 0.03 |
| PA1350 | PA1350 | 0.24 | 0.00 |
| PA1359 | PA1359 | 0.07 | 0.01 |
| PA1365 | PA1365 | 0.16 | 0.01 |
| PA1366 | PA1366 | 0.12 | 0.01 |
| PA1368 | PA1368 | 0.17 | 0.00 |
| PA1380 | PA1380 | 0.15 | 0.00 |
| PA1382 | PA1382 | 0.16 | 0.01 |
| PA1388 | PA1388 | 0.15 | 0.01 |
| PA1389 | PA1389 | 0.15 | 0.00 |
| PA1408 | PA1408 | 0.22 | 0.01 |
| PA1411 | PA1411 | 0.14 | 0.00 |
| PA1436 | PA1436 | 0.19 | 0.01 |
| PA1466 | PA1466 | 0.20 | 0.05 |
| PA1489 | PA1489 | 5.47 | 0.02 |
| PA1496 | PA1496 | 0.18 | 0.00 |
| PA1504 | PA1504 | 0.15 | 0.04 |
| PA1510 | PA1510 | 0.20 | 0.03 |
| PA1511 | PA1511 | 0.24 | 0.02 |
| PA1513 | PA1513 | 0.13 | 0.00 |
| PA1514 | PA1514 | 0.09 | 0.05 |
| PA1517 | PA1517 | 0.04 | 0.01 |
| PA1521 | PA1521 | 0.17 | 0.00 |
| PA1536 | PA1536 | 0.23 | 0.05 |
| PA1558 | PA1558 | 0.06 | 0.00 |
| PA1569 | PA1569 | 0.11 | 0.00 |
| PA1573 | PA1573 | 0.19 | 0.03 |
| PA1595 | PA1595 | 0.09 | 0.00 |
| PA1601 | PA1601 | 0.21 | 0.05 |
| PA1604 | PA1604 | 0.24 | 0.05 |
| PA1613 | PA1613 | 0.21 | 0.02 |
| PA1615 | PA1615 | 0.22 | 0.00 |
| PA1617 | PA1617 | 0.12 | 0.04 |
| PA1628 | PA1628 | 0.19 | 0.01 |
| PA1639 | PA1639 | 0.09 | 0.01 |
| PA1640 | PA1640 | 0.22 | 0.00 |
| PA1655 | PA1655 | 0.10 | 0.01 |
| PA1658 | PA1658 | 0.12 | 0.01 |
| PA1667 | PA1667 | 0.26 | 0.02 |
| PA1672 | PA1672 | 0.08 | 0.01 |
| PA1682 | PA1682 | 0.08 | 0.04 |
| PA1692 | PA1692 | 0.17 | 0.03 |
| PA1735 | PA1735 | 0.25 | 0.04 |
| PA1751 | PA1751 | 4.39 | 0.00 |
| PA1753 | PA1753 | 0.20 | 0.05 |
| PA1762 | PA1762 | 0.20 | 0.05 |
| PA1766 | PA1766 | 0.13 | 0.01 |
| PA1768 | PA1768 | 0.09 | 0.00 |
| PA1809 | PA1809 | 0.17 | 0.00 |
| PA1828 | PA1828 | 0.24 | 0.05 |
| PA1844 | PA1844 | 0.10 | 0.00 |
| PA1856 | PA1856 | 0.09 | 0.01 |
| PA1860 | PA1860 | 0.06 | 0.01 |
| PA1865 | PA1865 | 0.18 | 0.00 |
| PA1866 | PA1866 | 0.20 | 0.00 |
| PA1872 | PA1872 | 0.25 | 0.02 |
| PA1888 | PA1888 | 0.18 | 0.00 |
| PA1906 | PA1906 | 0.19 | 0.04 |
| PA1918 | PA1918 | 0.21 | 0.00 |
| PA1923 | PA1923 | 0.21 | 0.01 |
| PA1931 | PA1931 | 0.13 | 0.00 |
| PA1933 | PA1933 | 0.16 | 0.01 |
| PA1939 | PA1939 | 0.16 | 0.01 |
| PA1944 | PA1944 | 0.23 | 0.02 |
| PA1952 | PA1952 | 5.12 | 0.01 |
| PA1960 | PA1960 | 0.24 | 0.03 |
| PA1963 | PA1963 | 0.16 | 0.03 |
| PA1995 | PA1995 | 0.17 | 0.00 |
| PA1997 | PA1997 | 0.21 | 0.01 |
| PA2037 | PA2037 | 0.17 | 0.01 |
| PA2041 | PA2041 | 0.18 | 0.01 |
| PA2044 | PA2044 | 0.19 | 0.01 |
| PA2048 | PA2048 | 814.24 | 0.02 |
| PA2062 | PA2062 | 0.19 | 0.03 |
| PA2101 | PA2101 | 0.17 | 0.01 |
| PA2103 | PA2103 | 0.18 | 0.03 |
| PA2114 | PA2114 | 0.16 | 0.01 |
| PA2124 | PA2124 | 0.23 | 0.01 |
| PA2150 | PA2150 | 0.20 | 0.02 |
| PA2160 | PA2160 | 0.16 | 0.01 |
| PA2162 | PA2162 | 0.20 | 0.00 |
| PA2168 | PA2168 | 0.21 | 0.03 |
| PA2179 | PA2179 | 0.17 | 0.00 |
| PA2209 | PA2209 | 0.22 | 0.00 |
| PA2218 | PA2218 | 0.18 | 0.01 |
| PA2222 | PA2222 | 0.15 | 0.00 |
| PA2252 | PA2252 | 0.16 | 0.01 |
| PA2261 | PA2261 | 0.26 | 0.05 |
| PA2265 | PA2265 | 0.18 | 0.04 |
| PA2266 | PA2266 | 0.13 | 0.05 |
| PA2276 | PA2276 | 0.10 | 0.02 |
| PA2296 | PA2296 | 0.15 | 0.02 |
| PA2310 | PA2310 | 0.17 | 0.00 |
| PA2322 | PA2322 | 0.12 | 0.03 |
| PA2333 | PA2333 | 0.23 | 0.02 |
| PA2336 | PA2336 | 0.18 | 0.01 |
| PA2345 | PA2345 | 0.19 | 0.00 |
| PA2363 | PA2363 | 0.16 | 0.02 |
| PA2365 | PA2365 | 0.17 | 0.03 |
| PA2367 | PA2367 | 31.70 | 0.02 |
| PA2369 | PA2369 | 0.18 | 0.01 |
| PA2377 | PA2377 | 0.15 | 0.00 |
| PA2393 | PA2393 | 0.13 | 0.01 |
| PA2406 | PA2406 | 0.15 | 0.01 |
| PA2414 | PA2414 | 0.21 | 0.00 |
| PA2430 | PA2430 | 0.21 | 0.02 |
| PA2439 | PA2439 | 0.25 | 0.02 |
| PA2449 | PA2449 | 0.25 | 0.01 |
| PA2450 | PA2450 | 0.24 | 0.04 |
| PA2477 | PA2477 | 0.23 | 0.01 |
| PA2482 | PA2482 | 0.12 | 0.04 |
| PA2489 | PA2489 | 0.21 | 0.02 |
| PA2490 | PA2490 | 0.18 | 0.01 |
| PA2524 | PA2524 | 0.22 | 0.03 |
| PA2527 | PA2527 | 0.16 | 0.00 |
| PA2530 | PA2530 | 0.24 | 0.02 |
| PA2533 | PA2533 | 0.24 | 0.01 |
| PA2538 | PA2538 | 0.18 | 0.01 |
| PA2555 | PA2555 | 0.22 | 0.01 |
| PA2561 | PA2561 | 0.19 | 0.00 |
| PA2567 | PA2567 | 0.21 | 0.04 |
| PA2568 | PA2568 | 0.11 | 0.04 |
| PA2588 | PA2588 | 0.14 | 0.01 |
| PA2590 | PA2590 | 0.13 | 0.02 |
| PA2595 | PA2595 | 0.18 | 0.03 |
| PA2610 | PA2610 | 0.17 | 0.01 |
| PA2628 | PA2628 | 0.11 | 0.01 |
| PA2633 | PA2633 | 0.12 | 0.02 |
| PA2661 | PA2661 | 0.15 | 0.01 |
| PA2668 | #N/A | 0.19 | 0.03 |
| PA2676 | PA2676 | 0.10 | 0.00 |
| PA2684 | PA2684 | 0.22 | 0.01 |
| PA2692 | PA2692 | 0.13 | 0.00 |
| PA2698 | PA2698 | 0.16 | 0.03 |
| PA2708 | PA2708 | 0.19 | 0.04 |
| PA2711 | PA2711 | 0.17 | 0.00 |
| PA2712 | PA2712 | 0.16 | 0.00 |
| PA2714 | PA2714 | 0.19 | 0.01 |
| PA2720 | PA2720 | 19.62 | 0.00 |
| PA2727 | #N/A | 0.19 | 0.01 |
| PA2730 | PA2730 | 0.10 | 0.02 |
| PA2732 | PA2732 | 0.18 | 0.00 |
| PA2734 | PA2734 | 0.18 | 0.00 |
| PA2735 | PA2735 | 0.23 | 0.03 |
| PA2750a | PA2750a | 0.13 | 0.01 |
| PA2753 | PA2753 | 0.22 | 0.01 |
| PA2757 | PA2757 | 0.10 | 0.00 |
| PA2760 | PA2760 | 0.21 | 0.02 |
| PA2770 | PA2770 | 0.13 | 0.02 |
| PA2776 | PA2776 | 0.16 | 0.02 |
| PA2780 | PA2780 | 12.07 | 0.01 |
| PA2793 | PA2793 | 0.17 | 0.00 |
| PA2801 | PA2801 | 0.19 | 0.01 |
| PA2814 | PA2814 | 0.15 | 0.01 |
| PA2815 | PA2815 | 0.22 | 0.01 |
| PA2823 | PA2823 | 0.24 | 0.01 |
| PA2828 | PA2828 | 0.22 | 0.01 |
| PA2839 | PA2839 | 6.21 | 0.00 |
| PA2854 | PA2854 | 0.09 | 0.00 |
| PA2872 | PA2872 | 0.16 | 0.01 |
| PA2878 | PA2878 | 0.11 | 0.01 |
| PA2906 | PA2906 | 0.20 | 0.00 |
| PA2928 | PA2928 | 0.24 | 0.03 |
| PA2929 | PA2929 | 0.20 | 0.04 |
| PA2945 | PA2945 | 0.24 | 0.04 |
| PA2954 | PA2954 | 0.07 | 0.02 |
| PA2956 | PA2956 | 0.17 | 0.01 |
| PA2963 | PA2963 | 0.14 | 0.00 |
| PA2973 | PA2973 | 0.21 | 0.01 |
| PA3024 | PA3024 | 0.22 | 0.01 |
| PA3027 | PA3027 | 0.16 | 0.01 |
| PA3034 | PA3034 | 0.10 | 0.01 |
| PA3038 | PA3038 | 0.13 | 0.02 |
| PA3043 | PA3043 | 0.16 | 0.00 |
| PA3047 | PA3047 | 0.13 | 0.01 |
| PA3048 | PA3048 | 0.25 | 0.00 |
| PA3053 | PA3053 | 0.13 | 0.03 |
| PA3055 | PA3055 | 0.20 | 0.01 |
| PA3057 | PA3057 | 0.12 | 0.03 |
| PA3065 | PA3065 | 0.17 | 0.00 |
| PA3070 | PA3070 | 0.18 | 0.01 |
| PA3124 | PA3124 | 0.13 | 0.01 |
| PA3131 | PA3131 | 0.20 | 0.01 |
| PA3136 | PA3136 | 0.20 | 0.02 |
| PA3137 | PA3137 | 0.22 | 0.00 |
| PA3143 | PA3143 | 0.24 | 0.02 |
| PA3170 | PA3170 | 0.18 | 0.02 |
| PA3174 | PA3174 | 0.23 | 0.01 |
| PA3177 | PA3177 | 0.22 | 0.03 |
| PA3179 | PA3179 | 0.15 | 0.01 |
| PA3185 | PA3185 | 0.20 | 0.00 |
| PA3213 | PA3213 | 0.17 | 0.00 |
| PA3216 | PA3216 | 0.06 | 0.02 |
| PA3228 | PA3228 | 0.14 | 0.01 |
| PA3233 | PA3233 | 0.23 | 0.03 |
| PA3234 | PA3234 | 0.17 | 0.03 |
| PA3238 | PA3238 | 0.12 | 0.00 |
| PA3251 | PA3251 | 0.13 | 0.01 |
| PA3264 | PA3264 | 0.19 | 0.03 |
| PA3268 | PA3268 | 0.20 | 0.00 |
| PA3272 | PA3272 | 0.16 | 0.01 |
| PA3273 | PA3273 | 0.13 | 0.00 |
| PA3278 | PA3278 | 0.10 | 0.00 |
| PA3285 | PA3285 | 4.04 | 0.00 |
| PA3286 | PA3286 | 0.19 | 0.00 |
| PA3291 | PA3291 | 0.22 | 0.02 |
| PA3295 | PA3295 | 0.14 | 0.02 |
| PA3301 | PA3301 | 0.14 | 0.01 |
| PA3303 | PA3303 | 0.11 | 0.00 |
| PA3304 | PA3304 | 0.26 | 0.02 |
| PA3306 | PA3306 | 0.21 | 0.00 |
| PA3309 | PA3309 | 0.18 | 0.02 |
| PA3310 | PA3310 | 0.15 | 0.00 |
| PA3313 | PA3313 | 0.16 | 0.00 |
| PA3316 | PA3316 | 0.14 | 0.02 |
| PA3317 | PA3317 | 0.14 | 0.05 |
| PA3318 | PA3318 | 0.18 | 0.01 |
| PA3325 | PA3325 | 0.14 | 0.00 |
| PA3336 | PA3336 | 0.05 | 0.00 |
| PA3343 | PA3343 | 0.16 | 0.00 |
| PA3345 | PA3345 | 0.25 | 0.05 |
| PA3358 | PA3358 | 0.16 | 0.01 |
| PA3368 | PA3368 | 0.22 | 0.04 |
| PA3381 | PA3381 | 0.18 | 0.01 |
| PA3399 | PA3399 | 0.18 | 0.00 |
| PA3403 | PA3403 | 0.12 | 0.01 |
| PA3421 | PA3421 | 0.11 | 0.00 |
| PA3446 | PA3446 | 0.17 | 0.01 |
| PA3449 | PA3449 | 0.21 | 0.00 |
| PA3459 | PA3459 | 0.24 | 0.02 |
| PA3460 | PA3460 | 0.21 | 0.03 |
| PA3467 | PA3467 | 0.17 | 0.01 |
| PA3468 | PA3468 | 0.18 | 0.00 |
| PA3469 | PA3469 | 0.21 | 0.02 |
| PA3485 | PA3485 | 0.15 | 0.01 |
| PA3488 | PA3488 | 0.11 | 0.00 |
| PA3503 | #N/A | 0.12 | 0.04 |
| PA3504 | PA3504 | 0.16 | 0.01 |
| PA3508 | PA3508 | 0.25 | 0.03 |
| PA3513 | PA3513 | 0.14 | 0.00 |
| PA3516 | PA3516 | 0.18 | 0.01 |
| PA3522 | PA3522 | 0.17 | 0.00 |
| PA3532 | PA3532 | 0.20 | 0.02 |
| PA3566 | PA3566 | 0.16 | 0.00 |
| PA3568 | PA3568 | 0.20 | 0.01 |
| PA3586 | PA3586 | 0.22 | 0.01 |
| PA3588 | PA3588 | 0.21 | 0.03 |
| PA3592 | PA3592 | 0.23 | 0.03 |
| PA3597 | PA3597 | 0.26 | 0.02 |
| PA3605 | PA3605 | 0.21 | 0.03 |
| PA3612 | PA3612 | 0.16 | 0.00 |
| PA3614 | PA3614 | 0.20 | 0.01 |
| PA3615 | PA3615 | 0.22 | 0.00 |
| PA3664 | PA3664 | 0.17 | 0.00 |
| PA3674 | PA3674 | 0.24 | 0.04 |
| PA3676 | PA3676 | 0.20 | 0.00 |
| PA3688 | PA3688 | 0.18 | 0.01 |
| PA3698 | PA3698 | 0.20 | 0.00 |
| PA3699 | PA3699 | 0.05 | 0.04 |
| PA3710 | PA3710 | 0.15 | 0.00 |
| PA3714 | PA3714 | 0.16 | 0.00 |
| PA3716 | PA3716 | 0.21 | 0.01 |
| PA3730 | PA3730 | 0.13 | 0.01 |
| PA3731 | PA3731 | 0.07 | 0.00 |
| PA3732 | PA3732 | 0.11 | 0.00 |
| PA3755 | PA3755 | 0.15 | 0.00 |
| PA3768 | PA3768 | 0.14 | 0.00 |
| PA3772 | PA3772 | 0.23 | 0.01 |
| PA3774 | PA3774 | 0.22 | 0.01 |
| PA3776 | PA3776 | 0.14 | 0.00 |
| PA3780 | PA3780 | 0.07 | 0.02 |
| PA3789 | PA3789 | 0.15 | 0.01 |
| PA3794 | PA3794 | 0.09 | 0.04 |
| PA3795 | PA3795 | 0.18 | 0.00 |
| PA3797 | PA3797 | 0.16 | 0.00 |
| PA3800 | PA3800 | 0.06 | 0.00 |
| PA3801 | PA3801 | 0.06 | 0.04 |
| PA3825 | PA3825 | 0.19 | 0.00 |
| PA3871 | PA3871 | 0.15 | 0.01 |
| PA3881 | PA3881 | 0.26 | 0.04 |
| PA3882 | PA3882 | 0.15 | 0.02 |
| PA3888 | PA3888 | 0.23 | 0.04 |
| PA3890 | PA3890 | 0.16 | 0.01 |
| PA3892 | PA3892 | 0.10 | 0.00 |
| PA3893 | PA3893 | 0.20 | 0.00 |
| PA3897 | PA3897 | 0.20 | 0.03 |
| PA3905 | PA3905 | 41.25 | 0.03 |
| PA3908 | PA3908 | 0.21 | 0.01 |
| PA3919 | PA3919 | 0.14 | 0.00 |
| PA3920 | PA3920 | 0.19 | 0.00 |
| PA3924 | PA3924 | 0.12 | 0.01 |
| PA3928 | PA3928 | 0.08 | 0.04 |
| PA3932 | PA3932 | 0.22 | 0.00 |
| PA3939 | PA3939 | 0.21 | 0.01 |
| PA3949 | PA3949 | 0.23 | 0.03 |
| PA3951 | PA3951 | 0.15 | 0.00 |
| PA3952 | PA3952 | 0.20 | 0.01 |
| PA3960 | PA3960 | 0.16 | 0.02 |
| PA3963 | PA3963 | 0.15 | 0.02 |
| PA3992 | PA3992 | 0.22 | 0.01 |
| PA4016 | PA4016 | 0.20 | 0.00 |
| PA4037 | PA4037 | 0.20 | 0.00 |
| PA4039 | PA4039 | 0.12 | 0.00 |
| PA4061 | PA4061 | 0.21 | 0.02 |
| PA4069 | PA4069 | 0.07 | 0.00 |
| PA4072 | PA4072 | 0.14 | 0.00 |
| PA4080 | PA4080 | 0.00 | 0.01 |
| PA4096 | PA4096 | 0.07 | 0.02 |
| PA4108 | PA4108 | 0.16 | 0.04 |
| PA4112 | PA4112 | 0.24 | 0.02 |
| PA4120 | PA4120 | 0.17 | 0.00 |
| PA4121 | PA4121 | 0.23 | 0.01 |
| PA4126 | PA4126 | 0.21 | 0.00 |
| PA4131 | PA4131 | 0.08 | 0.00 |
| PA4132 | PA4132 | 0.05 | 0.00 |
| PA4136 | PA4136 | 0.09 | 0.01 |
| PA4143 | PA4143 | 0.15 | 0.00 |
| PA4145 | PA4145 | 0.20 | 0.02 |
| PA4150 | PA4150 | 0.11 | 0.04 |
| PA4157 | PA4157 | 5.22 | 0.00 |
| PA4166 | PA4166 | 0.19 | 0.01 |
| PA4182 | PA4182 | 0.23 | 0.01 |
| PA4186 | PA4186 | 0.17 | 0.01 |
| PA4189 | PA4189 | 0.24 | 0.01 |
| PA4193 | PA4193 | 0.13 | 0.03 |
| PA4198 | PA4198 | 0.19 | 0.00 |
| PA4219 | PA4219 | 0.19 | 0.04 |
| PA4222 | PA4222 | 0.21 | 0.03 |
| PA4223 | PA4223 | 0.22 | 0.00 |
| PA4288 | PA4288 | 0.20 | 0.03 |
| PA4290 | PA4290 | 0.14 | 0.00 |
| PA4317 | PA4317 | 0.11 | 0.02 |
| PA4330 | PA4330 | 6.77 | 0.00 |
| PA4332 | PA4332 | 0.06 | 0.01 |
| PA4334 | PA4334 | 0.20 | 0.00 |
| PA4342 | PA4342 | 0.20 | 0.00 |
| PA4346 | PA4346 | 0.10 | 0.02 |
| PA4348 | PA4348 | 0.07 | 0.00 |
| PA4358 | PA4358 | 0.09 | 0.00 |
| PA4361 | PA4361 | 0.26 | 0.05 |
| PA4369 | PA4369 | 0.09 | 0.04 |
| PA4372 | PA4372 | 0.16 | 0.00 |
| PA4390 | PA4390 | 0.14 | 0.01 |
| PA4391 | PA4391 | 0.28 | 0.04 |
| PA4394 | PA4394 | 0.06 | 0.02 |
| PA4400 | PA4400 | 0.16 | 0.00 |
| PA4436 | PA4436 | 0.11 | 0.01 |
| PA4438 | PA4438 | 0.11 | 0.01 |
| PA4454 | PA4454 | 0.17 | 0.00 |
| PA4475 | PA4475 | 0.15 | 0.01 |
| PA4478 | PA4478 | 0.09 | 0.02 |
| PA4489 | PA4489 | 0.21 | 0.00 |
| PA4498 | PA4498 | 36.27 | 0.00 |
| PA4499 | PA4499 | 84.11 | 0.04 |
| PA4513 | PA4513 | 0.23 | 0.03 |
| PA4514 | PA4514 | 0.13 | 0.00 |
| PA4517 | PA4517 | 0.25 | 0.05 |
| PA4518 | PA4518 | 0.14 | 0.03 |
| PA4521 | PA4521 | 0.17 | 0.01 |
| PA4534 | PA4534 | 0.21 | 0.01 |
| PA4539 | PA4539 | 0.23 | 0.01 |
| PA4541 | PA4541 | 0.16 | 0.00 |
| PA4574 | PA4574 | 0.12 | 0.04 |
| PA4577 | PA4577 | 0.17 | 0.00 |
| PA4579 | PA4579 | 0.22 | 0.00 |
| PA4583 | PA4583 | 0.18 | 0.00 |
| PA4589 | PA4589 | 0.21 | 0.00 |
| PA4591 | PA4591 | 0.18 | 0.00 |
| PA4608 | PA4608 | 0.22 | 0.02 |
| PA4612 | PA4612 | 0.16 | 0.00 |
| PA4617 | PA4617 | 0.24 | 0.01 |
| PA4618 | PA4618 | 0.15 | 0.00 |
| PA4619 | PA4619 | 0.23 | 0.01 |
| PA4627 | PA4627 | 0.14 | 0.00 |
| PA4631 | PA4631 | 0.12 | 0.01 |
| PA4632 | PA4632 | 0.14 | 0.01 |
| PA4649 | PA4649 | 0.16 | 0.02 |
| PA4651 | PA4651 | 0.24 | 0.00 |
| PA4653 | PA4653 | 0.19 | 0.05 |
| PA4654 | PA4654 | 0.24 | 0.00 |
| PA4657 | PA4657 | 0.22 | 0.02 |
| PA4658 | PA4658 | 0.16 | 0.00 |
| PA4690a | PA4690a | 0.24 | 0.00 |
| PA4691 | PA4691 | 0.23 | 0.03 |
| PA4692 | PA4692 | 0.17 | 0.00 |
| PA4701 | PA4701 | 0.18 | 0.00 |
| PA4705 | PA4705 | 0.22 | 0.02 |
| PA4712 | PA4712 | 0.23 | 0.01 |
| PA4716 | PA4716 | 0.20 | 0.01 |
| PA4717 | PA4717 | 0.19 | 0.00 |
| PA4724 | PA4724 | 0.17 | 0.01 |
| PA4724.1 | PA4724.1 | 0.07 | 0.05 |
| PA4734 | PA4734 | 0.18 | 0.05 |
| PA4739 | PA4739 | 0.15 | 0.00 |
| PA4754 | PA4754 | 0.10 | 0.02 |
| PA4779 | PA4779 | 0.25 | 0.01 |
| PA4792 | PA4792 | 0.15 | 0.00 |
| PA4794 | PA4794 | 0.09 | 0.05 |
| PA4799 | PA4799 | 0.06 | 0.01 |
| PA4801 | PA4801 | 0.17 | 0.03 |
| PA4831 | PA4831 | 0.26 | 0.02 |
| PA4833 | PA4833 | 0.18 | 0.00 |
| PA4834 | PA4834 | 0.16 | 0.01 |
| PA4843 | PA4843 | 0.18 | 0.01 |
| PA4844 | PA4844 | 0.17 | 0.00 |
| PA4852 | PA4852 | 0.09 | 0.02 |
| PA4871 | PA4871 | 0.22 | 0.03 |
| PA4882 | PA4882 | 0.16 | 0.01 |
| PA4896 | PA4896 | 0.27 | 0.05 |
| PA4903 | PA4903 | 0.15 | 0.04 |
| PA4909 | PA4909 | 0.16 | 0.00 |
| PA4912 | PA4912 | 0.21 | 0.05 |
| PA4915 | PA4915 | 0.25 | 0.04 |
| PA4916 | PA4916 | 0.17 | 0.00 |
| PA4917 | PA4917 | 0.08 | 0.00 |
| PA4925 | PA4925 | 0.15 | 0.04 |
| PA4940 | PA4940 | 0.11 | 0.01 |
| PA4950 | PA4950 | 0.21 | 0.00 |
| PA4972 | PA4972 | 0.09 | 0.00 |
| PA4981 | PA4981 | 0.23 | 0.00 |
| PA4985 | PA4985 | 0.24 | 0.03 |
| PA4987 | PA4987 | 0.25 | 0.02 |
| PA4993 | PA4993 | 0.20 | 0.03 |
| PA5008 | PA5008 | 5.65 | 0.01 |
| PA5022 | PA5022 | 0.21 | 0.00 |
| PA5027 | PA5027 | 0.19 | 0.01 |
| PA5029 | PA5029 | 0.17 | 0.03 |
| PA5030 | PA5030 | 0.23 | 0.02 |
| PA5037 | PA5037 | 0.06 | 0.00 |
| PA5046 | PA5046 | 0.16 | 0.00 |
| PA5047 | PA5047 | 0.17 | 0.00 |
| PA5076 | PA5076 | 0.23 | 0.05 |
| PA5088 | PA5088 | 0.17 | 0.04 |
| PA5102 | PA5102 | 0.12 | 0.01 |
| PA5113 | PA5113 | 0.25 | 0.05 |
| PA5123 | PA5123 | 0.12 | 0.04 |
| PA5136 | PA5136 | 0.19 | 0.03 |
| PA5139 | PA5139 | 0.12 | 0.00 |
| PA5165 | PA5165 | 0.23 | 0.02 |
| PA5179 | PA5179 | 0.19 | 0.00 |
| PA5182 | PA5182 | 0.19 | 0.02 |
| PA5185 | PA5185 | 0.08 | 0.02 |
| PA5186 | PA5186 | 0.23 | 0.03 |
| PA5187 | PA5187 | 0.19 | 0.01 |
| PA5207 | PA5207 | 0.17 | 0.00 |
| PA5208 | PA5208 | 0.21 | 0.01 |
| PA5209 | PA5209 | 0.20 | 0.00 |
| PA5210 | PA5210 | 0.21 | 0.03 |
| PA5220 | PA5220 | 0.25 | 0.01 |
| PA5226 | PA5226 | 0.26 | 0.03 |
| PA5233 | PA5233 | 0.08 | 0.01 |
| PA5236 | PA5236 | 0.11 | 0.00 |
| PA5238 | PA5238 | 0.18 | 0.02 |
| PA5254 | PA5254 | 0.25 | 0.02 |
| PA5264 | PA5264 | 0.16 | 0.00 |
| PA5273 | PA5273 | 0.18 | 0.01 |
| PA5283 | PA5283 | 0.24 | 0.02 |
| PA5303 | PA5303 | 16.02 | 0.00 |
| PA5307 | PA5307 | 0.14 | 0.01 |
| PA5317 | PA5317 | 0.17 | 0.00 |
| PA5329 | PA5329 | 0.16 | 0.00 |
| PA5340 | PA5340 | 0.16 | 0.01 |
| PA5346 | PA5346 | 0.07 | 0.00 |
| PA5362 | PA5362 | 0.10 | 0.00 |
| PA5377 | PA5377 | 0.11 | 0.02 |
| PA5378 | PA5378 | 0.15 | 0.01 |
| PA5383 | PA5383 | 0.08 | 0.01 |
| PA5388 | PA5388 | 0.24 | 0.01 |
| PA5392 | PA5392 | 0.16 | 0.00 |
| PA5393 | PA5393 | 0.23 | 0.01 |
| PA5396 | PA5396 | 0.08 | 0.01 |
| PA5397 | PA5397 | 0.23 | 0.05 |
| PA5412 | PA5412 | 0.25 | 0.02 |
| PA5428 | PA5428 | 0.11 | 0.05 |
| PA5435 | PA5435 | 0.11 | 0.01 |
| PA5440 | PA5440 | 0.10 | 0.00 |
| PA5455 | PA5455 | 0.09 | 0.01 |
| PA5458 | PA5458 | 0.21 | 0.00 |
| PA5471 | PA5471 | 0.13 | 0.02 |
| PA5474 | PA5474 | 0.19 | 0.00 |
| PA5487 | PA5487 | 0.18 | 0.00 |
| PA5508 | PA5508 | 0.14 | 0.02 |
| PA5515 | PA5515 | 0.10 | 0.00 |
| PA5524 | PA5524 | 0.11 | 0.01 |
| PA5526 | PA5526 | 0.16 | 0.01 |
| PA5527 | PA5527 | 0.14 | 0.02 |
| PA5529 | PA5529 | 0.17 | 0.00 |
| PA5530 | PA5530 | 0.15 | 0.02 |
| PA5540 | PA5540 | 0.18 | 0.01 |
| PA5542 | PA5542 | 0.19 | 0.04 |
| PA5545 | PA5545 | 0.20 | 0.00 |
| PA5547 | PA5547 | 0.15 | 0.00 |
| panB | PA4729 | 0.04 | 0.04 |
| panD | PA4731 | 0.05 | 0.00 |
| pbpG | PA0869 | 0.18 | 0.03 |
| pcaR | PA0155 | 0.18 | 0.00 |
| pcaT | PA0229 | 0.15 | 0.00 |
| pcnB | PA4727 | 0.17 | 0.00 |
| pcrV | PA1706 | 0.12 | 0.00 |
| pcs | PA3857 | 0.17 | 0.04 |
| pelA | PA3064 | 0.23 | 0.00 |
| pelD | PA3061 | 0.14 | 0.00 |
| pelF | PA3059 | 0.22 | 0.01 |
| pelG | PA3058 | 0.14 | 0.00 |
| pgi | PA4732 | 0.16 | 0.02 |
| phaC1 | PA5056 | 0.17 | 0.00 |
| phaC2 | PA5058 | 0.23 | 0.01 |
| phoP | PA1179 | 0.21 | 0.04 |
| phr | PA4660 | 0.21 | 0.00 |
| phuT | PA4708 | 0.19 | 0.01 |
| phzA1 | PA4210 | 0.02 | 0.01 |
| phzM | PA4209 | 0.14 | 0.01 |
| pilA | PA4525 | 0.06 | 0.01 |
| pilF | PA3805 | 0.19 | 0.00 |
| pilJ | PA0411 | 0.06 | 0.00 |
| pilP | PA5041 | 0.04 | 0.00 |
| pilQ | PA5040 | 0.25 | 0.02 |
| pilR | PA4547 | 0.10 | 0.00 |
| piv | PA4175 | 0.20 | 0.02 |
| plcH | PA0844 | 0.24 | 0.03 |
| plcR | PA0843 | 0.24 | 0.01 |
| pmbA | PA4472 | 0.15 | 0.01 |
| pmtA | PA0798 | 0.24 | 0.02 |
| ponA | PA5045 | 0.22 | 0.02 |
| ppk | PA5242 | 0.17 | 0.00 |
| pppA | PA0075 | 0.05 | 0.03 |
| pqqE | PA1989 | 0.11 | 0.00 |
| pqqH | PA1990 | 0.20 | 0.01 |
| prc | PA3257 | 0.19 | 0.01 |
| prtR | PA0611 | 17.55 | 0.04 |
| pslH | PA2238 | 0.10 | 0.00 |
| pslM | PA2243 | 0.21 | 0.02 |
| pstA | PA5367 | 0.12 | 0.01 |
| ptsP | PA0337 | 0.23 | 0.00 |
| ptxS | PA2259 | 0.12 | 0.00 |
| purL | PA3763 | 0.06 | 0.00 |
| purM | PA0945 | 0.10 | 0.03 |
| pvdH | PA2413 | 0.23 | 0.00 |
| pvdT | PA2390 | 0.22 | 0.01 |
| pykA | PA4329 | 0.17 | 0.00 |
| pyrE | PA5331 | 0.19 | 0.04 |
| pys2 | PA1150 | 0.21 | 0.04 |
| qor | PA0023 | 0.09 | 0.02 |
| qscR | PA1898 | 0.17 | 0.02 |
| recA | PA3617 | 0.18 | 0.00 |
| recG | PA5345 | 0.16 | 0.00 |
| recQ | PA3344 | 0.19 | 0.01 |
| rfaD | PA3337 | 0.17 | 0.00 |
| rluC | PA2975 | 0.24 | 0.02 |
| rmf | PA3049 | 0.12 | 0.05 |
| rnr | PA4937 | 0.19 | 0.00 |
| rplS | PA3742 | 0.12 | 0.02 |
| rpsG | PA4267 | 0.25 | 0.03 |
| sbcD | PA4281 | 0.24 | 0.02 |
| sbp | PA0283 | 0.11 | 0.02 |
| selD | PA1642 | 0.17 | 0.01 |
| smpB | PA4768 | 0.21 | 0.03 |
| sodM | PA4468 | 0.15 | 0.00 |
| soxA | PA5418 | 0.19 | 0.01 |
| speC | PA4519 | 0.20 | 0.05 |
| speD | PA0654 | 0.11 | 0.01 |
| speE | PA1687 | 0.10 | 0.00 |
| spuA | PA0297 | 0.16 | 0.00 |
| spuC | PA0299 | 0.17 | 0.03 |
| spuD | PA0300 | 0.19 | 0.04 |
| spuE | PA0301 | 0.22 | 0.01 |
| spuI | PA0296 | 0.20 | 0.02 |
| tag | PA0010 | 0.21 | 0.03 |
| thiI | PA5118 | 0.16 | 0.00 |
| thrC | PA3735 | 0.24 | 0.02 |
| thrH | PA1757 | 0.11 | 0.00 |
| tig | PA1800 | 0.17 | 0.01 |
| tpbA | PA3885 | 0.16 | 0.01 |
| trkA | PA0016 | 0.13 | 0.00 |
| ubiA | PA5358 | 0.20 | 0.02 |
| vqsM | PA2227 | 93.92 | 0.05 |
| wspA | PA3708 | 0.19 | 0.02 |
| wspC | PA3706 | 0.16 | 0.01 |
| xcpW | PA3098 | 0.21 | 0.01 |
| xdhB | PA1523 | 0.13 | 0.03 |
| xseB | PA4042 | 0.18 | 0.00 |
| yfiB | PA1119 | 0.09 | 0.03 |

**Table S2. The expression of some pyoverdine synthesis related genes.**

| **Gene name** | **Log_2_*FC*** | ***P* value** | **Product** |
| --- | --- | --- | --- |
| *fpvA* | -3.2 | 2.77E-79 | Ferric pyoverdine receptor |
| *fpvB* | -2.9 | 1.13E-154 | Second ferric pyoverdine receptor |

*FC*, fold change.

**Table S3. The contacting residues of PqsH and zapE.**

| CR of PqsH | CR of zapE | Interaction type |
| --- | --- | --- |
| Arg168.NH1 | Asp19.OD2 | Salt bridge |
| Arg168.NH1 | Ser197.OG | Hydrogen bond |
| Arg168.NH2 | Gly198.O | Hydrogen bond |
| Arg332.NH1/NH2 | Ser197.O | Hydrogen bond |
| Lys333.Nz | Asp200.OD1/OD2 | Salt bridge |
| Trp336.O | Arg204.NH2/NE | Hydrogen bond |
| Arg340.NH1/NH2 | Pro14.O | Hydrogen bond |
| Arg340.NH1 | Phe16.O | Hydrogen bond |
| Arg340.NE | Gln208.OE1 | Hydrogen bond |
| Ser364.OG | Gln353.O | Hydrogen bond |
| Arg367.NH1/NH2 | Glu351.OE1/OE2 | Salt bridge |
| Arg367.NH1 | Glu356.OE1 | Salt bridge |
| Arg371.NH1 | Glu356.OE2 | Salt bridge |
| Arg371.NH2 | Thr359.OG1 | Hydrogen bond |

CR: contacting residue.

**Table S4. Strains and plasmids used in this study.**

| **Strain or plasmid** | **Genotype or relevant characteristics** | **Sources** |
| --- | --- | --- |
| ***P. aeruginosa* series strains** | | |
| PAO1 | Wild type strain | Laboratory store |
| ΔPA0222 | PA0222 in frame deletion strain | This study |
| ΔPA1112 | PA1112 in frame deletion strain | This study |
| ΔPA2345 | PA2345 in frame deletion strain | This study |
| ΔPA3797 | PA3797 in frame deletion strain | This study |
| ΔPA4438 | PA4438 in frame deletion strain | This study |
| Δ*psl*Δ*pel* | *pslBCD* and *pelA* deletion strain | This study |
| Δ*pqsH* | *pqsH in frame deletion strain* | This study |
| PAO1-gfp | Wild type strain with mono copy of GFP inserted into genome | This study |
| PAO1-mCherry | Wild type strain with mono copy of mCherry inserted into genome | This study |
| ΔPA0222-gfp | PA0222 deletion mutant with mono copy of GFP inserted into genome | This study |
| ΔPA1112-gfp | PA1112 deletion mutant with mono copy of GFP inserted into genome | This study |
| ΔPA2345-gfp | PA2345 deletion mutant with mono copy of GFP inserted into genome | This study |
| ΔPA3797-gfp | PA3797 deletion mutant with mono copy of GFP inserted into genome | This study |
| ΔPA4438-gfp | PA4438 deletion mutant with mono copy of GFP inserted into genome | This study |
| PAO1::*zapE* | *zapE* with mono copy at the CTX phage att site on PAO1 chromosome | This study |
| Δ*zapE*::*zapE* | *zapE* with mono copy at the CTX phage att site onΔ*zapE* chromosome | This study |
| PAO1/p*PpqsA-gfp* | p*PpqsA*-gfp in PAO1, Ap^r^, Gm^r^ | This study |
| ΔPA4438/p*PpqsA-gfp* | p*PpqsA*-gfp inΔPA4438, Ap^r^, Gm^r^ | This study |
| PAO1::*zapE*/p*PpqsA-gfp* | p*PpqsA*-gfp in PAO1::*zapE*, Ap^r^, Gm^r^ | This study |
| Δ*zapE*::*zapE*/p*PpqsA-gfp* | p*PpqsA*-gfp in Δ*zapE*::*zapE*, Ap^r^, Gm^r^ | This study |
| PAO1/pUCP20 | pUCP20 in PAO1, Carb^r^ | This study |
| ΔPA4438/pUCP20 | pUCP20 in ΔPA4438, Carb^r^ | This study |
| ΔPA4438/pPA4438 | pUCP20 with PA4438, Carb^r^ | This study |
| ΔPA4438/p*eczapE* | pUCP20 with *zapE* from *E. coli* K12 (*eczapE*), Carb^r^ | This study |
| ΔPA4438/pK72A | pUCP20 with K72A, Carb^r^ | This study |
| PAO1-gfp/p20T | pHerd20T in PAO1-gfp, Carb^r^ | This study |
| PAO1-mCherry/p20T | pHerd20T in PAO1-mCherry, Carb^r^ | This study |
| ΔPA4438-gfp/p20T | pHerd20T in ΔPA4438-gfp, Carb^r^ | This study |
| ΔPA4438*-gfp*/pPA4438 | pHerd20T with PA4438 in ΔPA4438-gfp, Carb^r^ | This study |
| ΔPA4438/p20T-gfp | pHerd20T-gfp in ΔPA4438, Carb^r^ | This study |
| ΔPA4438/p20T-PA4438-gfp | pHerd20T-PA4438-gfp in ΔPA4438, Carb^r^ | This study |
| PAK | Wild type strain | Laboratory store |
| PAKΔ*zapE* | *zapE* in frame deletion strain | This study |
| PAKΔ*zapE/*p*zapE* | pUCP20 with *zapE*, Carb^r^ | This study |
| PA14 | Wild type strain | Laboratory store |
| PA14Δ*zapE* | *zapE* in frame deletion strain | This study |
| PA14Δ*zapE/*p*zapE* | pUCP20 with *zapE*, Carb^r^ | This study |
| ***Escherichia coli*** | | |
| DH5α | *E. coli*, F^-^, φ 80d*lacZ* ΔM15, Δ(*lacZYA* -*argF*)U169, *deoR*, *recA_1_*, *endA_1_*, *hsdR_17_* (rK^-^, mK^+^), *phoA*, *supE_44_*, *λ^-^*, *thi -1*, *gyrA_96_* , *relA_1_* | Sangon |
| K12 | *E. coli* reference strain | Novagen |
| BL21(DE3) | *fhuA2 [lon] ompT gal* (λ DE3) [*dcm*] *∆hsdS* | Sangon |
| **Plasmids** | | |
| pBT20 | Mariner transposon mutagenesis plasmid, Gm^r^/Ap^r^ | From Professor Luyan Z. Ma |
| pHerd20T | *E. coli-P. aeruginosa* shuttle plasmid containing arabinose inducible P_BAD_ promoter, Ap^r^ | From Professor Luyan Z. Ma |
| miniCTX-lacZ | Gene delivery vector for inserting genes at the CTX phage att site on *P.* *aeruginosa* chromosome | Laboratory store |
| pUCP20 | *E. coli-P. aeruginosa* shuttle plasmid, Ap^r^ | Laboratory store |
| pBF13 | Helper plasmid, Ap^r^ | Laboratory store |
| pRK600 | Helper plasmid, Cm^r^ | Laboratory store |
| pK18-Gm-mob*sacB* | Suicide knock-out plasmid, Gm^r^ | Laboratory store |
| pET28a | Protein expression vector with his tag, Kan^r^ | Laboratory store |
| pET28a-*zapE* | pET28a with *zapE*, Kan^r^ | This study |
| pET28a-K72A | pET28a with K72A, Kan^r^ | This study |
| pMAL-c5x | Protein expression vector with MBP tag, Ap^r^ | Laboratory store |
| pMAL-c5x-*pqsH* | pMAL-c5x with *pqsH*, Ap^r^ | This study |
| pminiCTX-Tn7-GFP | Tn7 transposon plasmid with GFP tag, Gm^r^ | Laboratory store |
| pminiCTX-Tn7-mCherry | Tn7 transposon plasmid with mCherry tag, Gm^r^ | Laboratory store |
| p20T-*gfp* | pHerd20T with *gfp*, Ap^r^ | This study |
| p20T-*zapE*-*gfp* | pHerd20T with *zapE*-*gfp* fusion gene, Ap^r^ | This study |
| p*zapE* | pUCP20 with *zapE*, Ap^r^ | This study |
| p*eczapE* | pUCP20 with *zapE* from *E. coli* K12, Ap^r^ | This study |
| pK72A | pUCP20 with ZapE_K72A_, Ap^r^ | This study |
| pP*pqsA*-gfp | pUCP22Not-P*pqsA*-RBS-CDS-RNaseIII-*gfp*(ASV)-T0-T1, Ap^r^, Gm^r^ | Laboratory store |

**Table S5. Primers used in this study.**

| Names | Sequences | |
| --- | --- | --- |
| UPA0222-F | | agctcggtacccggggatccCACGCGGGTCAGGGAGGAGA |
| UPA0222-R | | CGACGCGCAGCGCGCGGAACACTCCCACAGCCGATTG |
| DPA0222-F | | CAATCGGCTGTGGGAGTGTTCCGCGCGCTGCGCGTCG |
| DPA0222-R | | cgacggccagtgccaagccttATCGTCCGCGAAGTGGACAACGT |
| PA0222-TF | | ATCGACAAGACCCGTATCGC |
| PA0222-TR | | TATCTGCAGGGGTTGTTGGAA |
| UPA1112-F | | agctcggtacccggggatccGGGCGCCTGATGAAGGCG |
| UPA1112-R | | TCAAGCGCAACCAAGGCTGAAGGGCGTTCTCTCCTTCCCCAAGCG |
| DPA1112-F | | CGCTTGGGGAAGGAGAGAACGCCCTTCAGCCTTGGTTGCGCTTGA |
| DPA1112-R | | cgacggccagtgccaagccttAACCGTCGCGCCGAAAGCC |
| PA1112-TF | | ATCGCTATATCAATGCCAAGGGCAC |
| PA1112-TR | | GGAAAAACTGCTCACCGAACTGGTC |
| UPA2345-F | | agctcggtacccggggatccGGGCGTCGTTCTCGATGAGG |
| UPA2345-R | | GAGGGCTGCCGCCCGGTGGGAGTTCCTTCGACGATCC |
| DPA2345-F | | GGATCGTCGAAGGAACTCCCACCGGGCGGCAGCCCTC |
| DPA2345-R | | cgacggccagtgccaagccttCCAACGTGCGCCTCGACCC |
| PA2345-TF | | AACAGTTTCTGCTGGTCCGCG |
| PA2345-TR | | TCGCGGGAAGCGATCGA |
| UPA3797-F | | agctcggtacccggggatccCTGTCCCACGACGAGTTGTATCCG |
| UPA3797-R | | ACGCGGAACACGCCGTCGTCAGATCGCGCATAGCTTTTCCG |
| DPA3797-F | | CGGAAAAGCTATGCGCGATCTGACGACGGCGTGTTCCGCGT |
| DPA3797-R | | cgacggccagtgccaagccttTCCAGGAATTCAGGGCATCGCC |
| PA3797-TF | | ACCTGGACGACGTGGCCAT |
| PA3797-TR | | CGCCGAACCGAGAATCGAAT |
| UPA4438-F | | agctcggtacccggggatccCAAGGCGCGCCGCATC |
| UPA4438-R | | CTTTCTCGTTTCCGCCCCGGAGATCGGTAGCAATGCAACGAAAACG |
| DPA4438-F | | CGTTTTCGTTGCATTGCTACCGATCTCCGGGGCGGAAACGAGAAAG |
| DPA4438-R | | cgacggccagtgccaagccttCGCGCCTGGCCGAGGAG |
| UpqsH-F | | AGCTCGGTACCCGGGGATCCTAAGGGGTTGACAGGAGCGGG |
| UpqsH-R | | CGACCAAGACGCCACTGGCACCGTTGCTCCTTAGCAGCGG |
| DpqsH-F | | CCGCTGCTAAGGAGCAACGGTGCCAGTGGCGTCTTGGTCG |
| DpqsH-R | | cgacggccagtgccAAGCTTCCATCAGGACGACATCCGGCTT |
| UPAK*zapE*-F | | agctcggtacccggggatccCAAGGCGCGCCGCATC |
| UPAK*zapE*-R | | CTTTCTCGTTTCCGCCCCGGAGATCGGTAGCAATGCAACGAAAACG |
| DPAK*zapE*-F | | CGTTTTCGTTGCATTGCTACCGATCTCCGGGGCGGAAACGAGAAAG |
| DPAK*zapE*-R | | cgacggccagtgccaagccttCGCGCCTGGCCGAGGAG |
| UPA14*zapE*-F | | agctcggtacccggggatccCAAGGCGCGCCGCATC |
| UPA14*zapE*-R | | CTTTCTCGTTTCCGCCCCGGAGATCGGTAGCAATGCAACGAAAACG |
| DPA14*zapE*-F | | CGTTTTCGTTGCATTGCTACCGATCTCCGGGGCGGAAACGAGAAAG |
| DPA14*zapE*-R | | cgacggccagtgccaagccttCGCGCCTGGCCGAGGAG |
| PA4438-TF | | AATGCCTTTGGCCTGTGGC |
| PA4438-TR | | ATCAAGCCGATCAACGCCA |
| *zapE*-F | | atgggatctgataagaattcATGACGCCCCTCGAGCGCTACC |
| *zapE*-R | | cgacggccagtgccaagcttTCAGGGCCGGTGCGGACG |
| CTX-*zapE*-F | | gaggtcgacggtatcgataagctCATCGCCACCCCATTCATCGCC |
| CTX-*zapE*-R | | tcccccgggctgcaggaattTCAGGGCCGGTGCGGACG |
| T-CTX-F | | GGGTTTTCCCAGTCACGACGTTGTA |
| T-CTX-R | | ACCGGCGCGCGTAATACGAC |
| Ec*zapE*-F | | atgggatctgataagaattcATGCAAAGCGTTACCCCAACAT |
| Ec*zapE*-R | | cgacggccagtgccaagcttTTAACCCGCCAAATGCTCG |
| Pa-Rnd1-F | | GGCCACGCGTCGACTAGTACSNNNNNNNSNSSSGCG |
| BT20-Rnd1-R | | TATAATGTGTGGAATTGTGAGCGG |
| Pa-Rnd2-F | | GGCCACGCGTCGACTAGTAC |
| BT20-Rnd2-R | | ACAGGAAACAGGACTCTAGAGG |
| Tn-AD-1 | | GATCGGAAGAGCACACGTCT |
| Tn-AD-2 | | ACACTCTTTCCCTACACGACGCTCTTCCGATCT |
| Tn-Rnd1-F | | ACACTCTTTCCCTACACGACGCTCTTCCGATCTCTGGAAGGCGAGCATCGTTTGTTC |
| Tn-Rnd1-R | | GTGACTGGAGTTCAGACGTGTGCTCTTCCGATCTTTCTGTATGGAACGGGATGCGTCTAAA |
| Tn-Rnd2-F | | AATGATACGGCGACCACCGAGATCTACACTCTTTCCCTACACGACGCTCTTCCGATC*T |
| Tn-Rnd2-R | | CAAGCAGAAGACGGCATACGAGAT[index]GTGACTGGAGTTCAGACGTGTGCTCTTCCGATCT |
| *gfp*-F | | atgggatctgataagaattcATGCGTAAAGGAGAAGAACTTTTC |
| *gfp*-R | | cgacggccagtgccaagcttTTATTTGTATAGTTCATCCATGCCAT |
| *zapE*-*gfp*-F1 | | atgggatctgataagaattcATGACGCCCCTCGAGCGCTACC |
| *zapE*-*gfp*-R1 | | CAGTGAAAAGTTCTTCTCCTTTACGGGGCCGGTGCGGACGC |
| *zapE*-*gfp*-F2 | | GCGTCCGCACCGGCCCCGTAAAGGAGAAGAACTTTTCACTG |
| *zapE*-*gfp*-R2 | | cgacggccagtgccaagcttTTATTTGTATAGTTCATCCATGCCAT |
| 28a-*zapE*-F | | GCAAATGGGTCGCGGATCCGAATTCATGACGCCCCTCGAGCGCTACC |
| 28a-*zapE*-R | | GGTGCTCGAGTGCGGCCGCAAGCTTTCAGGGCCGGTGCGGACG |
| MAL-*pqsH*-F | | tcacatatgtccatgggcggccgcATGACCGTTCTTATCCAGGGGG |
| MAL-*pqsH*-R | | tatttaattacctgcagggaattcCTACTGTGCGGCCATCTCACC |
| K72A-F | | CGTCGGCCGTGGCGCGACCTACCTGGTG |
| K72A-R | | CACCAGGTAGGTCGCGCCACGGCCGACG |


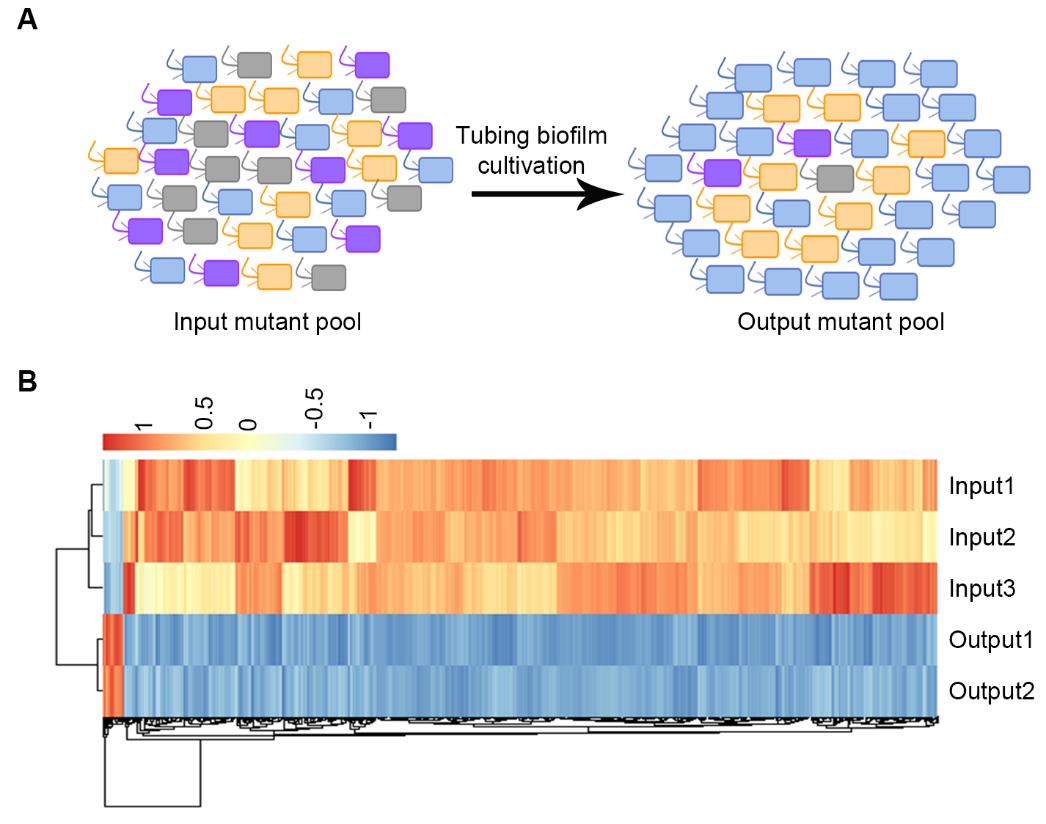


**Figure S1.** The integrated Tn-seq biofilm experiment in *P. aeruginosa* PAO1. (A) Schematic depicting changes in mutant abundance during tubing biofilm cultivation of PAO1 transposon insertion mutant library. These mutant abundance changes can be detected by Tn-seq analysis of the inoculum input mutant pool and tubing biofilm derived output mutant pool. The mutants with reduced biofilm formation ability were in purple and grey color, mutants which have no defect in biofilm formation ability was in orange color, and mutants that could form hyper-biofilm were in blue color. (B) Heatmap plot of the potential biofilm genetic determinants in Inputs and Outputs.


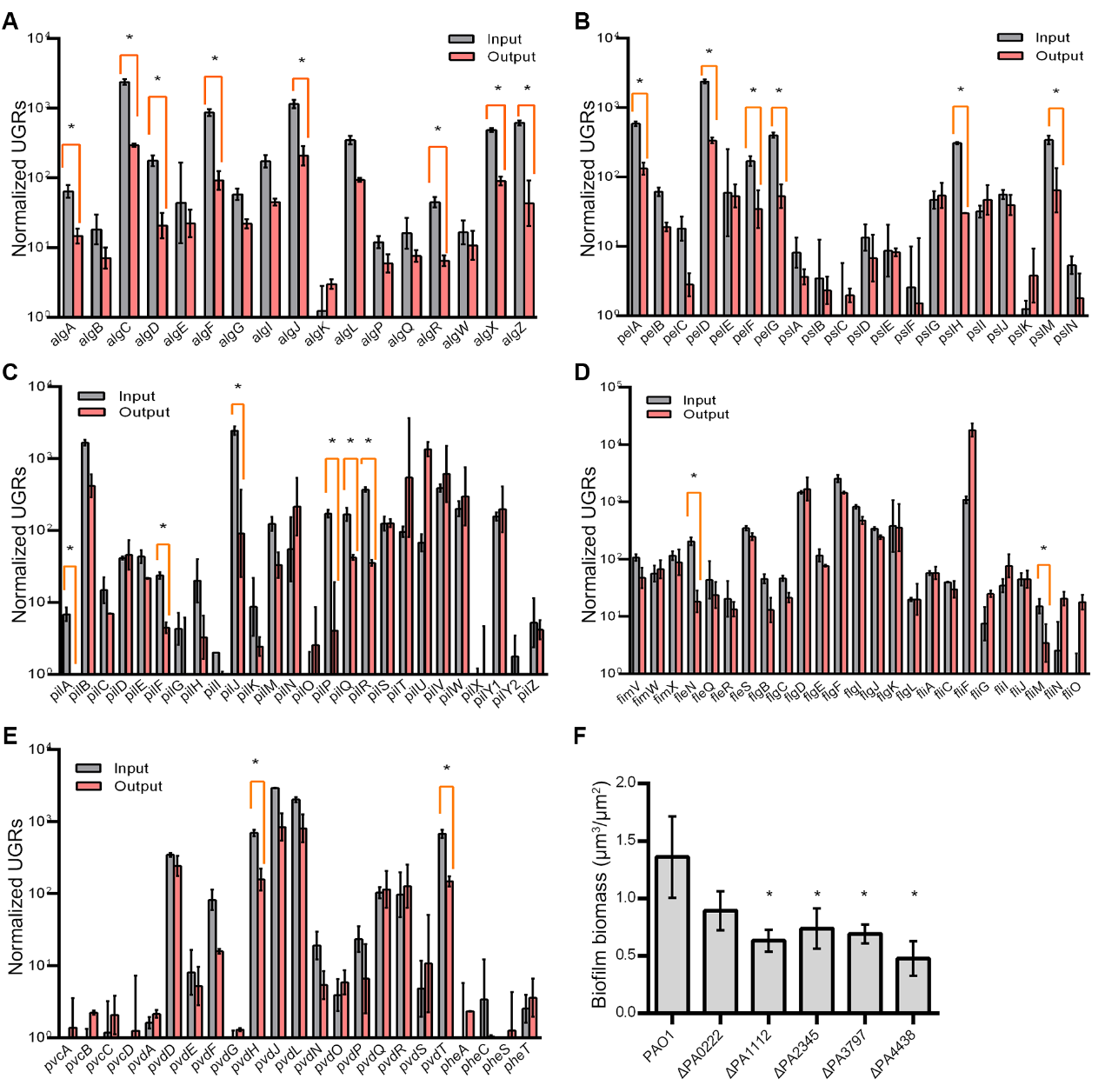


**Figure S2.** The number of mutants with insertion in well-characterized biofilm essential genes were decreased in output pools compared with input pools. Approximately half exopolysaccharides related insertion mutants lost their abundance during the biofilm formation, which is in consistence with their key roles in biofilm formation. The gene cluster of (A) alginate biosynthesis and (B) Pel and Psl biosynthesis reduced their abundance in biofilms, among which 47% and 30% showed statistical significance, respectively. The insertion mutants with disrupted pili (C) or flagella (D) showed less abundant in the biofilms output mutant pools comparing with the input mutant pools. The pyoverdine and phenazine metabolism (E) were also identified for their significant contribution in the formation of tubing biofilms. (F) The biofilm biomass of PAO1, ΔPA0222, ΔPA1112, ΔPA2345, ΔPA3797 and ΔPA4438. *, *P*≤0.05).


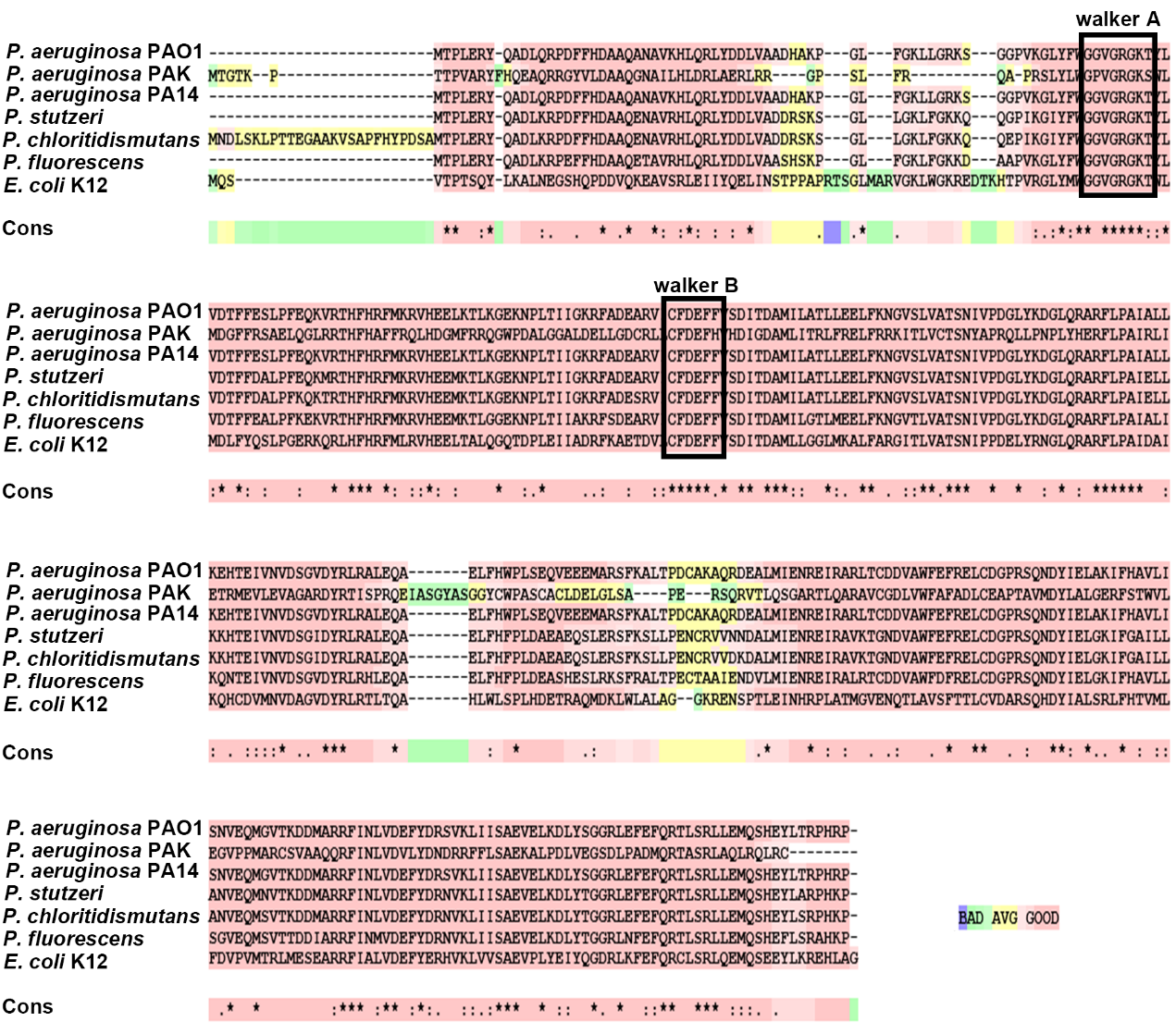


**Figure S3.** ZapE is highly conserved in different *Pseudomonas* species and *E. coli*. The alignment of ZapE primary structure of *P. aeruginosa* PAO1, PAK and PA14, *P. stutzeri*, *P. chloritidismutans* and *P. fluorescens* and *E. coli* K12. ZapE of above strains contain putative walker A and walker B sites. The conserved amino acids residues were shown in light and dark pink color. The alignment was made by T-coffee Version_11.00.


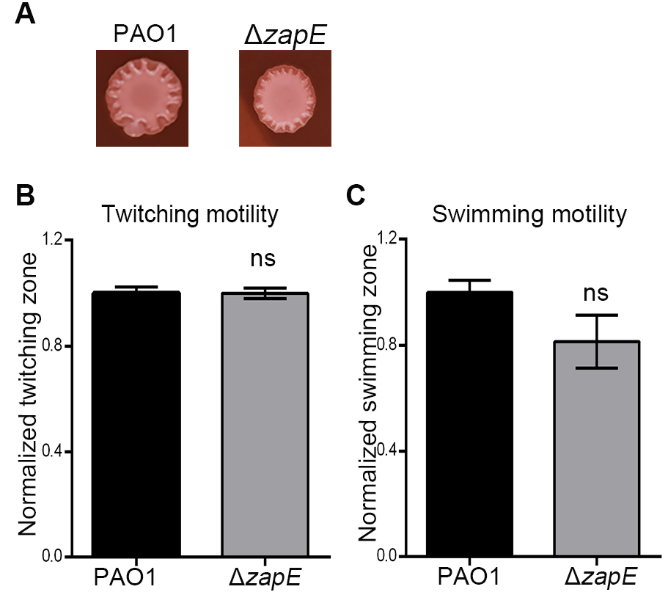


**Figure S4.** The phenotypes contributing to biofilm formation showed no significant difference between PAO1 and Δ*zapE*. (A) The colony morphology of Δ*zapE* and PAO1 on Congo red plates, which indicated the exopolysaccharides production. (B) The twitching zone of Δ*zapE* and PAO1. (C) The swimming zone of Δ*zapE* and PAO1.


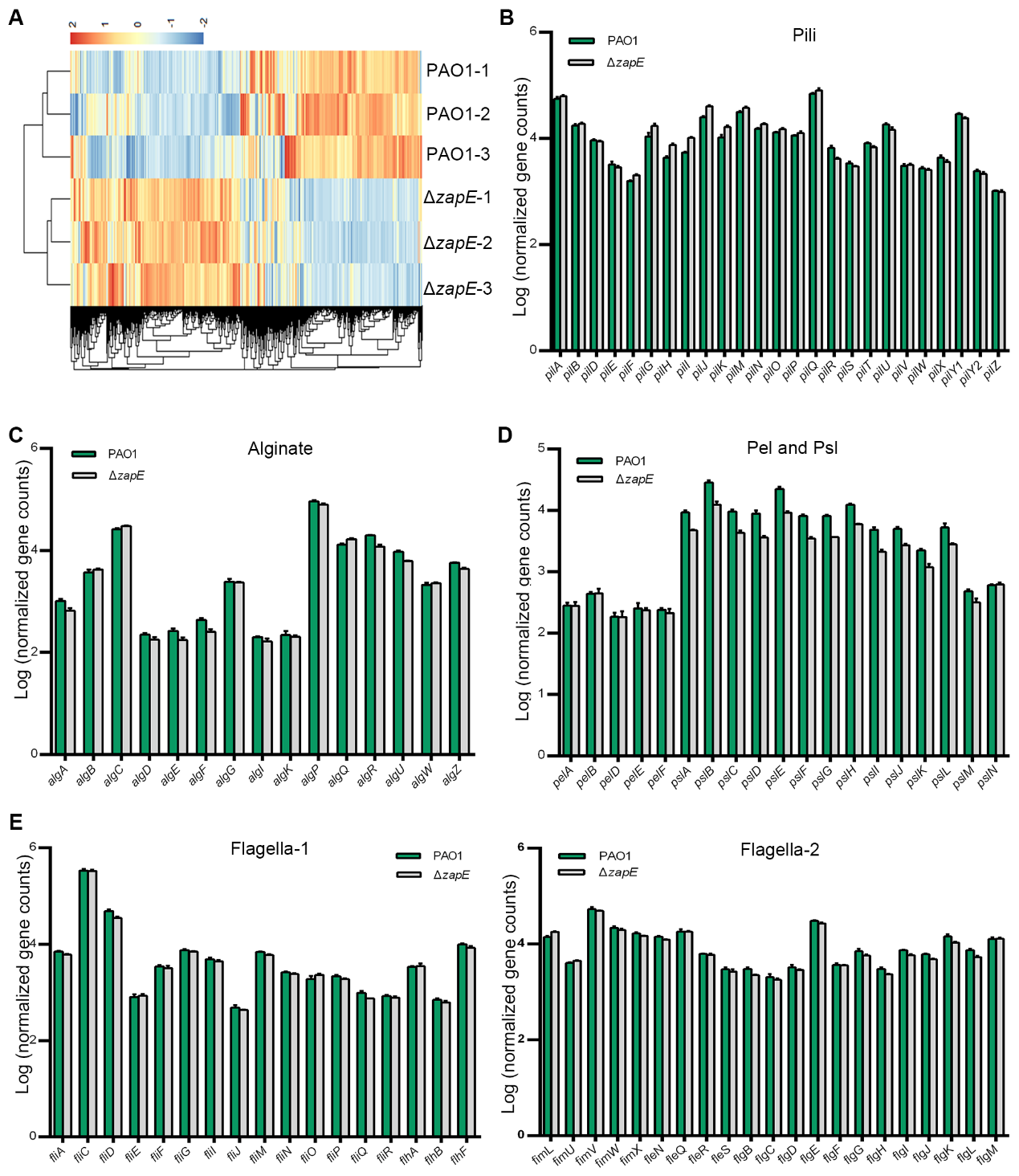


**Figure S5.** The genes encoding well-known biofilm structural factors such as exopolysaccharides, pili and flagella were expressed at similar level between PAO1 and Δ*zapE*. (A) Heatmap clustering of significant expression genes between PAO1 and Δ*zapE*. The expression of genes related to (B) pili, (C) alginate, (D) pel and psl, and (E) flagella.


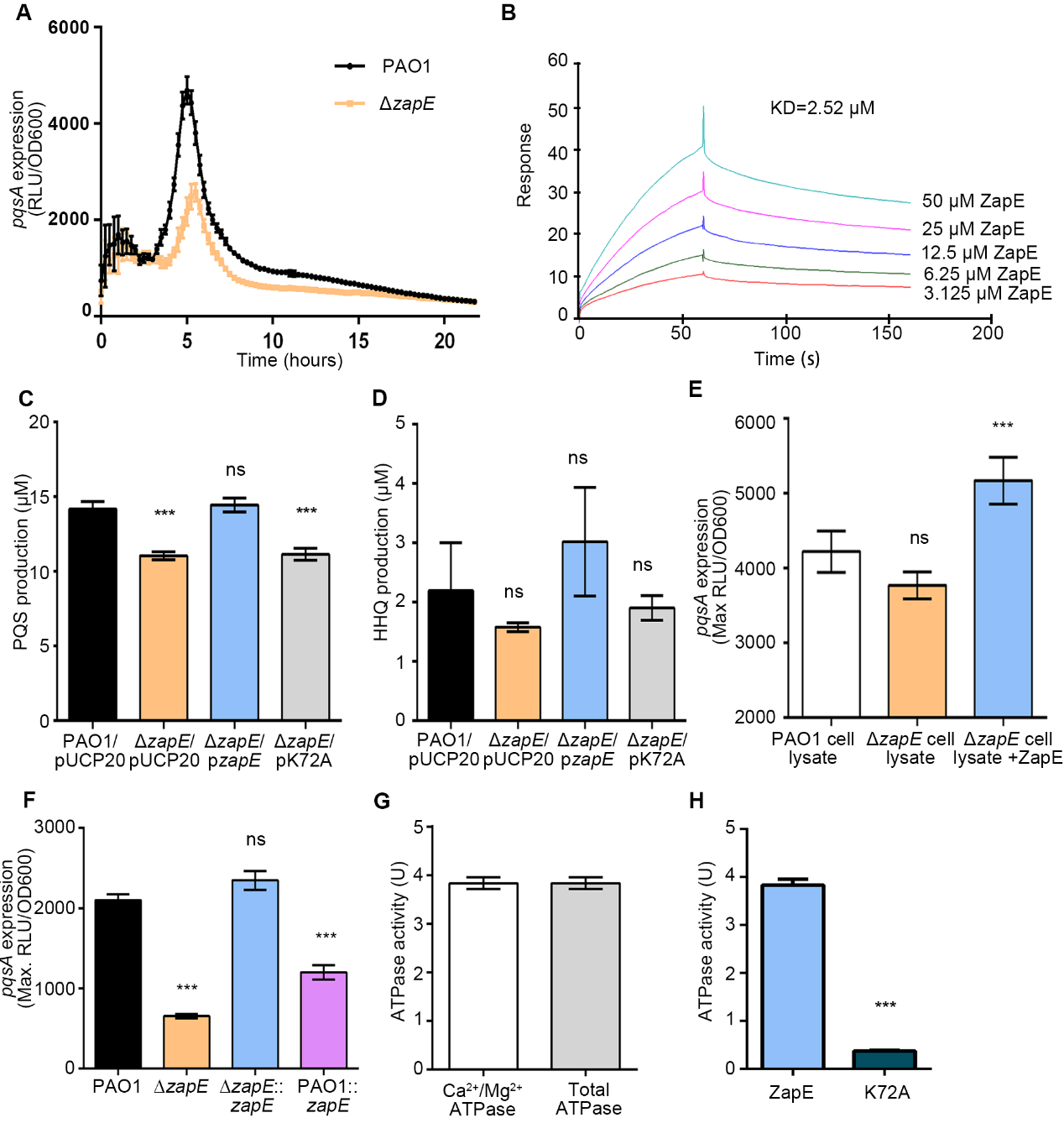


**Figure S6.** ZapE is an Ca^2+^/Mg^2+^ ATPase, which is required for the conversion of HHQ to PQS. (A) The *pqsA* expression curve of PAO1 and Δ*zapE*. (B) Direct binding of ZapE to PqsH. (C-D) The HHQ and PQS production of PAO1/pUCP20, Δ*zapE*/pUCP20, Δ*zapE*/p*zapE* and Δ*zapE*/pK72A. (E) The maximum *pqsA* expression of PAO1 with PAO1 cell lysate or PAO1 with same amount of Δ*zapE* cell lysate or PAO1 with same amount of Δ*zapE* cell lysate plus exogenously added recombinant ZapE at 1 μM. (F) The maximum *pqsA* expression of PAO1, Δ*zapE*, Δ*zapE*::*zapE* and PAO1::*zapE*. (G-H) The ATPase activity of ZapE and K72A. ***, *P*<0.001; ns, no significant.


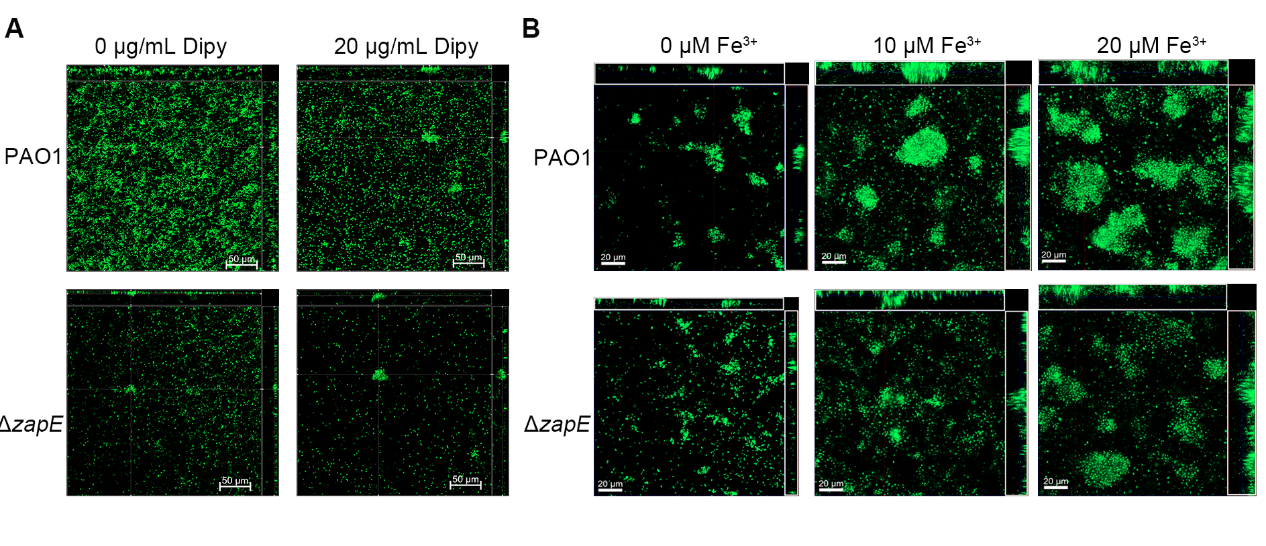


**Figure S7.** The biofilm morphology of PAO1 and Δ*zapE* under iron limited condition (20 μg/mL Dipy) (A), and under 10 μM and 20 μM ferric iron conditions (B). Scale bar, 50 μm.

**
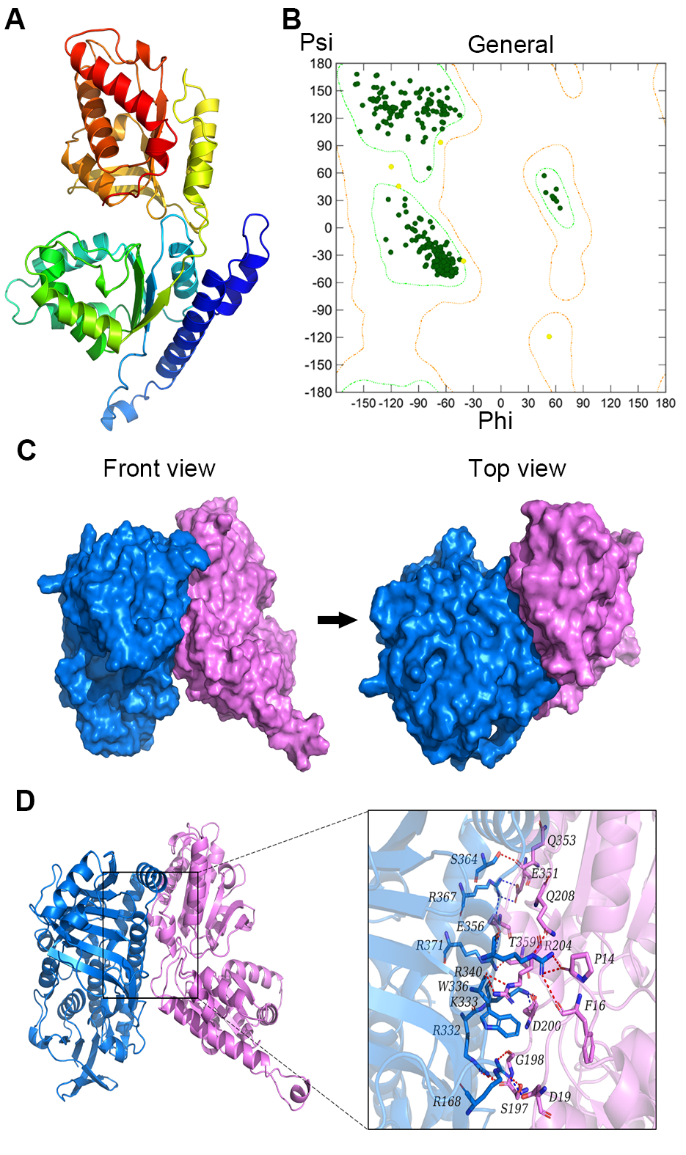
**

**Figure S8.** The three-dimensional structure of ZapE and the binding model of ZapE with PqsH. (A) The 3D structure of ZapE, which was constructed by AlphaFold. (B) The Ramachandran plot of ZapE. In Ramachandran plot, dark green dots represent the residues in favored regions; yellow dots represent the residues in allowed regions, red cross represent the residues in irrational regions, showing 100% residues were located in the allowed regions, which suggested that the 3D structure of ZapE is reasonable. (C) The surface binding model of ZapE with PqsH. (D) The 3D binding model of ZapE with PqsH. ZapE was in violet color, PqsH was in marine color. The residues in ZapE were shown as violet sticks, and the residues in PqsH were shown as marine sticks. The red dashes represent hydrogen bond interaction, and the blue dashes represent salt bridge.
